# Yeast-fermented feed improves high-concentrate diet-induced mastitis in dairy goats by regulating rumen microbiota

**DOI:** 10.3389/fmicb.2025.1582314

**Published:** 2025-06-26

**Authors:** Keyi Wu, Xiaochun Sun, Jiawen Xu, Zhihang Guan, Weijie Yuan, Lijuan Bao, Yihong Zhao, Ruping Shan, Hui Chen, Caijun Zhao, Xiaoyu Hu, Yunhe Fu, Dacheng Liu, Naisheng Zhang

**Affiliations:** ^1^Department of Gynecology, China-Japan Union Hospital of Jilin University, Changchun, Jilin, China; ^2^Department of Clinical Veterinary Medicine, College of Veterinary Medicine, Jilin University, Changchun, Jilin, China; ^3^College of Veterinary Medicine, Inner Mongolia Agricultural University, Hohhot, Inner Mongolia, China

**Keywords:** yeast-fermented feed, mastitis, rumen microbiota, high concentrate diet, LPS

## Abstract

Mastitis is a crucial disease that restricts the development of the dairy industry. In production practice, long-term high-concentrate diet (HCD) is often employed to boost milk yield. However, this can lead to rumen microbiota disorder and eventually results in mastitis. Microbial fermented feed has drawn increasing attention due to its abundant functions, safety, and effectiveness. Yeast, as a widely used fungus, has excellent fermentation performance and a variety of beneficial physiological functions. In this study, we investigated the therapeutic effect of yeast fermented feed (YFF) on mastitis in dairy goats induced by ruminal dysbiosis. Twenty-four dairy goats in late lactation, with an initial body weight of 34.65 ± 5.46 kg, were selected for the study. The total experimental period lasted 55 days. In the control group, a roughage-to-concentrate ratio (F:C) of 7:3 was consistently provided. For the subacute ruminal acidosis (SARA) group, eight dairy goats were given a 5:5 F:C diet free of charge for 10 days prior to the start of the experiment to ensure their adaptation to the diet. Subsequently, these eight dairy goats were fed an HCD consisting of 30% roughage and 70% mixed concentrate until the end of the experiment. For the SARA+Y group, eight dairy goats were fed the same diet as the SARA group from the beginning of the experiment until day 45. After that, 80 to 100 grams of YFF per goat were added to the diet daily for 10 days until the end of the experiment. In this study, adding YFF on the basis of HCD alleviated mastitis by restoring the function of the rumen barrier and regulating the imbalance of rumen microbiota. It alleviated the symptoms of SARA, reduced the levels of LPS in the rumen, serum, and mammary glands, and reduced the levels of proinflammatory cytokines. Specifically, it was reflected in restoring the function of the blood-milk barrier, limiting the inflammatory response, and reducing oxidative stress. In conclusion, these results suggest that supplementation with YFF alleviates mastitis induced by ruminal microbiota disturbance due to feeding HCD in several ways. This finding paves the way for a new approach and method to address mastitis in ruminant animals. It not only helps enhance the health level of ruminants but also plays a positive role in improving breeding efficiency. At the same time, it provides a strong guarantee for enhancing the quality of dairy products and lays a solid foundation for the sustainable development of the dairy industry.

## 1 Introduction

The dairy industry, as an important segment of the food industry, has witnessed significant progress in recent years. Consequently, enhancing production efficiency and reducing costs has become an inevitable trend in the development of the dairy industry. Cow mastitis is a crucial factor constraining the development of the dairy industry as it can impede the industry’s progress in multiple ways (Wenjin et al., 2020). Mastitis causes inflammation and swelling of the mammary glands, leading to a substantial decline in milk yield (Jing-Jing et al., 2017). Additionally, it increases the bacterial content and somatic cell count (SCC) in milk, affecting milk quality and reducing its market competitiveness (Huizhi et al., 2022). Currently, the mainstream treatment for mastitis in dairy cows is still antibiotic therapy. However, this can lead to excessive antibiotic residues in milk and bacterial resistance (Garcia et al., 2023). Therefore, from the perspectives of safety, economy, and efficiency, it is of great significance to explore new methods for the prevention and treatment of mastitis and fundamentally and effectively control the occurrence of mastitis.

In addition to bacterial infection as traditionally understood, a variety of factors are also regarded as important causes of mastitis. Various metabolic diseases, such as SARA (Xiaoyu et al., 2022) and ketosis (Ruibo et al., 2024), can lead to mastitis in dairy cows. SARA, which is a common metabolic disorder in dairy herds and has an impact on milk production in female cows (Meng et al., 2015), refers to a situation where the ruminal pH drops to an abnormal range (for example, the rumen pH of dairy cows is below 5.6 for more than 3 h per day) due to the accumulation of volatile fatty acids (VFA) in the rumen and the reduced ruminal buffering capacity in ruminant animals over an extended period (Elmhadi et al., 2022). The main cause of SARA is a lack of crude fiber or excessive concentrate in the diet (Plaizier et al., 2008). Under such circumstances, microorganisms in the rumen will rapidly consume digestible carbohydrates and produce a large amount of VFA. During SARA, a low rumen pH induces the release of lipopolysaccharide (LPS) from Gram-negative bacteria (Gozho et al., 2007). At the same time, the disruption of the ruminal epithelial barrier promotes the translocation of LPS into the bloodstream (Jiang et al., 2020), inducing endotoxemia, which in turn induces systemic inflammation, including mastitis (Wu et al., 2023). Moreover, SARA is also accompanied by disorder of the ruminal microbiota (Fu et al., 2022). The rumen of ruminants is an efficient fermentation system that degrades plant-derived cellulose and hosts a large number of microorganisms involved in the digestion and absorption of various nutrients. Therefore, we can consider the ruminal microbiota of ruminants similar to the gut microbiota of monogastric animals.

The gut microbiota consists of a diverse and large number of microbial groups, including bacteria, fungi, viruses, and so on (Wang et al., 2023). These microorganisms form a complex ecosystem in the intestine and have a symbiotic relationship with the host (Adak and Khan, 2019). The normal gut microbiota plays a crucial role in maintaining the body’s health (Agus et al., 2021). For instance, it helps in digesting food, synthesizing vitamins (Guetterman et al., 2022), and regulating the immune system (Fung et al., 2017). The host influences the composition of the gut microbiota, and in turn, the gut microbiota influences the host’s response (Sales and Reimer, 2023). Once the gut microbiota is disrupted, it will lead to the occurrence of various diseases, such as immunological diseases (Hans Ulrich et al., 2020), metabolic disorders (Kruttika et al., 2019), intestinal diseases (Ni et al., 2017) and so on. The gut microbiota can be adjusted through dietary intervention to restore its balance (Caijun et al., 2023a).

Probiotics are a class of active microorganisms that are beneficial to the host (Sanders et al., 2019). Supplementing probiotics can regulate the balance of the intestinal microbiota (Spencer et al., 2021) and improve disease symptoms (Caijun et al., 2021). For example, in the treatment of irritable bowel syndrome (Vivek et al., 2023) and inflammatory bowel disease (Zhen-Ping et al., 2024), certain probiotic preparations have demonstrated some efficacy. Yeasts are unicellular fungi that are widely dispersed in nature and possess significant applications in fields such as food, wine, and medicine (Ahasanul et al., 2020). As a type of probiotic, it has been proven to play a role in regulating gut microbiota (Seon-Kyun et al., 2019) and enhancing immunity (Yang et al., 2020). The main components of the yeast cell wall are glucans and mannan (Jinjing et al., 2018). The β-glucan in the cell wall of *Saccharomyces cerevisiae* can stimulate intestinal mucous cells to secrete mucus and increase the thickness of the intestinal mucus layer (Zhenyu et al., 2021). *Saccharomyces cerevisiae* can promote the expression of tight junction (TJ) proteins between intestinal epithelial cells and enhance the TJ of intestinal epithelial cells (Wen-Yang et al., 2020), thereby preventing harmful substances from entering the body through the intercellular space. In addition, yeast can improve the body’s resistance to pathogenic bacteria, reduce the invasion of pathogenic bacteria, and enhance the body’s ability to clear pathogenic bacteria (Karolina et al., 2020). A recent study has also shown that the addition of yeast has a beneficial effect on protecting liver health (Shendong et al., 2025).

In recent years, microbial fermented feed has drawn increasing attention from researchers (Ali Assar et al., 2019). It can not only improve the palatability of feed (Lin et al., 2019), enhance the growth performance of animals (Lv et al., 2022), but also improve the utilization rate of feed (Guo et al., 2022) and reduce the content of anti-nutritional factors in feed (Yuan et al., 2017). Therefore, we have proposed a yeast fermentation preparation, which is fermented from highly active yeast. Its main active components are glucan, mannan, and so on. In the present study, we investigated the therapeutic effect of supplementing with fermented feed on mastitis in dairy goats induced by ruminal dysbiosis. We hypothesized that YFF could alleviate HCD-induced mastitis and ruminal dysbiosis in dairy goats.

First of all, we discovered that long-term HCD induced SARA in dairy goats, which led to mastitis. This was manifested as the breakdown of the blood-milk barrier, activation of inflammatory pathways, and an increase in the level of oxidative stress. In addition, long-term HCD also caused ruminal damage and ruminal microbiota disorder in dairy goats. However, adding YFF to the diet of SARA dairy goats significantly improved mastitis, ruminal damage, and dysbiosis. These results strongly support the key role of YFF in ameliorating mastitis and ruminal dysbiosis.

## 2 Materials and methods

### 2.1 Animal ethics

All animal care and experimental procedures were approved by the Laboratory Animal Management and Ethics Committee of Jilin University and complied with the institution’s Laboratory Animal Use and Welfare Guidelines (20230867).

### 2.2 Animals and experimental design

Twenty-four dairy goats in late lactation (with an average body weight of 34.65 ± 5.46 kg, having similar lactation days and body weight) were selected for the experiment. In the control group, a roughage to concentrate ratio (F:C) of 7:3 was consistently provided. Eight dairy goats in the SARA group were provided with a 5:5 F:C diet offered ad libitum, that is, a 1:1 mixture of control diet and HCD diet, for 10 days prior to the start of the experiment to ensure the adaptation of dairy goats to the diet. Subsequently, these eight dairy goats were fed an HCD consisting of 30% roughage and 70% mixed concentrate until the end of the experiment (Meijuan et al., 2023). For the SARA+Y group, eight dairy goats were fed the same diet as the SARA group from the beginning of the experiment until day 45. After that, 80–100 g of YFF per goat was added to the diet per day for 10 days until the end of the experiment. Throughout the experiment, dairy goats were fed at 7:00 and 18:00 daily. They had free access to water during the experiment. All feed formulations are presented in Table 1.

**Table 1:** Ingredients and chemical composition of the experimental diet for goats.

Items (% of diet)	Diet	
	Control	HCD
Ingredients (% of DM)		
Oat grass hay	47.00	17.00
Alfalfa hay	13.00	8.50
Corn	30.50	64
Soybean meal	6.90	7.90
Salt	0.60	0.60
Limestone	0.50	0.50
Premix^a^	1.50	1.50
Nutrient composition		
DM (%)	88.71	87.45
ME (MJ/Kg of DM)	8.31	10.05
CP (% of DM)	11.33	11.33
NDF (% of DM)	41.24	23.14
NFC (% of DM)^b^	39.18	59.24
NFC/NDF	0.95	2.56
Calcium (% of DM)	0.56	0.40
Phosphorus (% DM)	0.27	0.28

a The premix provided the following per kg of diet: VA 120,000 IU, VD 50,000 IU, VE 700 IU, Nicotinic acid 450 mg, Cu 650 mg, Fe 400 mg, Mn 600 mg, Zn 1,000 mg, I 45 mg, Se 30 mg, Co 20 mg.

b % NFC = 100 - (% CP + % ash + % EE + % NDF).

The content of crude protein (CP) in feed was analyzed according to the China National Standard (GB/T 6432-2018) {CAA, 2018 #196}. The content of Ca in feed was analyzed according to the China National Standard (GB/T 6436-2018) {China Grain Wuhan Scientific Research & Design Institute Co., 2018 #197}. The content of P in feed was analyzed according to the China National Standard (GB/T 6437-2018) {Testing, 2018 #198}. The content of neutral detergent fiber in feed was analyzed according to the China National Standard (GB/T 20806-2006) {SIchuan Willtest Technology Co., 2022 #199}.

### 2.3 Production of YFF

Formulation of fermentation substrate: 15% wheat bran, 12% sprayed corn peel, 16% corn, 10% rice bran meal, 8% DDGS, 27% corn germ meal and 12% soybean meal. The fermentation strains are *Saccharomyces cerevisiae XR4* strain and *Kluyveromyces marxianus BC* strain, which are independently isolated and have independent intellectual property rights. The two strains are mixed in a ratio of 1:1 to make fermentation broth (1.5 × 10^8^ cfu/g), inoculated at a dose of 10% of the solid substrate mass, and a certain amount of water is added to make the initial water content of the fermentation substrate 38–40%.

Solid-state stacking fermentation is carried out in the fermentation workshop with a stacking height of 60–65 cm. During the fermentation process, the material temperature is recorded once every 3 h. When the fermentation time reaches 24 h and the temperature reaches above 40°C, the material is turned once. After the turning is completed, the stacking fermentation continues. The whole fermentation process is 72 h. After the fermentation is completed, low-temperature drying, crushing and bagging are carried out, and the preparation of yeast culture is completed. Its nutritional indicators are: crude protein ≥20.0%; crude ash ≤9.0%; moisture ≤12.0%; viable count ≥10^6^ cfu/g; neutral detergent fiber ≤34.0%; acid detergent fiber ≤20.0%. Active ingredient indicators are: mannan ≥0.35%; β-glucan ≥0.50%.

### 2.4 Determination of the number of viable yeasts

The plate pouring method was employed. 5 g of sample was weighed and placed in a triangular flask containing 45 mL of sterilized normal saline. Then, it was thoroughly shaken and mixed well. Use a micropipette to draw 1 mL and inject it into a test tube containing 9 mL of sterilized normal saline. Perform vortex shaking for approximately 1 min to obtain a bacterial solution with a concentration of 10^−1^. Multiple dilutions are carried out in this manner. Respectively draw 1 mL of 10^−5^ and 10^−6^ gradient bacterial solutions into sterile petri dishes. For each gradient, make three parallel dilutions. Subsequently, pour it into the rose bengal agar medium that has been treated by an autoclave and cooled to 45°C. Thoroughly mix it well. Wait for the plate to coagulate. Invert it and place it in a constant temperature incubator at 30°C for 48 h. Subsequently, perform colony counting.

### 2.5 Measurement of β-glucan and mannan

After drying at 55°C, 0.5 g fermented feed was weighed and placed into the test tube in three parallel replicates. 3 mL of deionized water was added to it and was shaken thoroughly. Collect the washing solution. Repeat this process five times and combine the washing solutions. Add 1 g of 0.4–0.6 mm glass beads to the washing solution. Then, add 2 mL of phosphate buffer with a pH of 8.0. Perform the treatment on a vortex mixer at a vibration speed of 4,000 rpm/min for 15 min on a 3-min cycle for a total of five cycles. The glass beads were collected from the treated sample solution and washed with 1 mL of deionized water, repeated five times, the wash solution was collected and combined with the sample solution, and the resulting liquid was left standing at 55°C for 24 h to evaporate water. This was followed by treatment with a 72% sulfuric acid solution. At the same time, different weights of β-glucan and mannan were weighed and hydrolyzed by sulfuric acid to make the calibration curve. The contents of β-glucan and mannan in each sample and corrected polysaccharide were determined by liquid chromatography-differential refractive detector. The calibration curve was made with the peak area as the ordinate and the concentration of corrected polysaccharide as the abscissa to obtain the regression equation. The peak area of the sample was substituted into the regression equation to calculate the content of β-glucan and mannan in the diet.

### 2.6 SCC

Milk goat teats were sterilized using lint dipped in 70% ethanol, and the first three handloads of milk were discarded. The samples were collected and transferred to sterile 50 mL sample bottles containing potassium dichromate. For the detection of SCC (Fossomatic 5000, FOSS).

### 2.7 Measurement of body temperature, pulse rate, and respiratory rate

After the establishment of the model, the body temperature, pulse rate, and respiratory rate of dairy goats were measured. The body temperature was measured rectally using a thermometer. The pulse rate was recorded by auscultation with a stethoscope. The respiratory rate was detected by observing the number of chest and abdominal fluctuations per minute in quiescent dairy goats.

### 2.8 Blood biochemical analysis

Blood samples from dairy goats were collected using a vacuum tube with thrombin, and subsequently blood biochemical analysis was performed using a commercial kit (DiAvTest, Russia) and an automatic biochemical analyzer (CS-T240, Dirui Industrial Co., LTD., China).

### 2.9 Complete blood count

At the conclusion of modeling, blood was sampled from the tail vein and collected in EDTA anticoagulant tubes for complete blood count.

### 2.10 LPS analysis

The levels of LPS in samples of rumen fluid, serum, and mammary glands were detected by a chromogenic endpoint assay (Chinese Horseshoe Crab Reagent Manufactory Co., Ltd., Xiamen, China). According to the manufacturer’s instructions, the minimum detection limit is 0.1 EU/mL for rumen fluid and 0.01 EU/mL for serum and mammary gland.

### 2.11 Histological analysis

The histological changes of the mammary tissue were evaluated by performing hematoxylin and eosin (H&E) staining. Mammary tissues from different treatment groups were fixed in 4% paraformaldehyde for 48 h and then processed into 5 μm sections. These sections were deparaffinized and hydrated using xylene and ethanol successively, and then stained with H&E. Subsequently, the histological changes were examined using light microscopy (Olympus, Tokyo, Japan). Mammary sections were scored according to established criteria (Caijun et al., 2021). The histological score was determined by assessing the extent of acinar structure destruction (0 indicating no signs of destruction, 1 indicating slight destruction, 2 indicating moderate destruction, and 3 indicating severe structure destruction) as well as the degree of inflammatory cell infiltration (0 representing no cell infiltration, 1 representing slight infiltration, 2 representing moderate infiltration, and 3 representing severe infiltration). Rumen pathological injury was scored using a scoring system that included two categories (Dai et al., 2017): severity of epithelial injury (graded 0–3, from absent to mild) and the extent of inflammatory cell infiltrate (graded 0–3, from none or rare to transmural).

### 2.12 MPO activity assay

To evaluate the degree of neutrophil infiltration in mammary tissues among different treatment groups, the activity of MPO was measured. A 10% tissue homogenate was prepared, and the MPO activity was determined in accordance with the manufacturer’s instructions.

### 2.13 Cytokines measurement by ELISA

To quantify the levels of TNF-α and IL-1β in mammary and rumen tissues, mammary tissue samples were collected from different treatment groups and homogenized in PBS to prepare 10% tissue homogenates. The resulting samples were then centrifuged at 12,000 × g and 4°C for 10 min, and the supernatants were carefully collected. Subsequently, the concentrations of TNF-α and IL-1β in the supernatants were determined using commercially available ELISA kits following the manufacturer’s instructions.

### 2.14 Western blotting (WB) analysis

The total protein samples of mammary and rumen tissue were collected by using the Tissue Protein Extract ACT (Thermo Fisher Scientific, USA). The protein concentrations were determined with the BCA Protein Assay Kit (Thermo Fisher Scientific, USA). Subsequently, target proteins were separated by 10% or 12% SDS-PAGE according to their molecular size. After being treated with methanol, they were immobilized onto 0.45 μm PVDF membranes. After blocking with 5% skim milk for 3 h at room temperature, the PVDF membranes were incubated overnight at 4°C with specific primary antibodies (ZO-1, occludin, Claudin-3, IκB, p-IκB, p65, p-p65, NLRP3, ASC, IL-1β, p38, p-p38 and β-actin), which were diluted to a concentration of 1:1,000. After being washed with TBST three times for 20 min each time interval, the PVDF membranes were then treated with secondary antibodies (Goat anti-rabbit IgG and Goat anti-mouse IgG) at a dilution of 1:10,000 for 2 h at room temperature. Finally, protein bands were visualized using the ECL plus western blotting Detection System.

### 2.15 Rumen fluid total DNA extraction and 16S rRNA sequencing

The Fast DNA Spin Kit for Soil (MP Biomedicals, USA) was utilized to extract total microbial DNA from rumen fluid, while the V3-V4 hypervariable region of bacterial 16S rRNA was amplified using specific primers (515F-806R). PCR reactions were performed in triplicate and the resulting PCR products were extracted from 2% agarose gels and purified with the AxyPrep DNA Gel Extraction kit (Axygen Biosciences, Union City, CA, USA) following the manufacturer’s instructions. Quantification was carried out using a Quantus fluorometer (Promega, USA). The purified amplicons were pooled in equimolar amounts and subjected to paired-end sequencing on an Illumina MiSeq PE300 platform/NovaSeq PE250 platform (Illumina, San Diego, USA) following the standard protocols established by Majorbio Bio-Pharm Technology Co. Ltd. (Shanghai, China). Sequencing libraries were generated using TruSeq DNA PCR-Free Sample Preparation Kit (Illumina, USA) following manufacturer’s recommendations and index codes were added. The library quality was assessed on the Qubit@ 2.0 Fluorometer (Thermo Scientific) and Agilent Bioanalyzer 2100 system. At last, the library was sequenced on an Illumina NovaSeq platform and 250 bp paired-end reads were generated. Operational taxonomic units (OTUs) with a 97% similarity cutoff were clustered using UPARSE version 7.1, and any chimeric sequences were identified and subsequently removed. The representative sequence of each OTU was taxonomically classified using RDP Classifier version 2.2 against the 16S rRNA database, employing a confidence threshold of 0.7. Alpha diversity is applied in analyzing the complexity of species diversity for a sample through six indices, including Observed-species, Chao1, Shannon, Simpson, ACE, Good-coverage. All the indices in our samples were calculated with QIIME (Version 1.7.0). Principal Coordinate Analysis (PCoA) was employed to discern the microbial structure, while Linear discriminant analysis Effect Size (LEfSe) was utilized to identify bacterial taxa that exhibited differential enrichment across various treatment groups. Spearman correlation analysis was performed to reveal the relationship between different microbial taxa and host parameters using the Wekemo Bioincloud. Additionally, alpha diversity analysis encompassed the computation of Shannon, Chao1, and ace indices. A Wilcoxon rank-sum test (FDR < 0.05) was used to identify the difference in the differential bacterial taxa between the two groups.

### 2.16 Materials

Ampicillin (Cat# A5354), neomycin (Cat# N6386), metronidazole (Cat# 16677) and vancomycin (Cat# V2002) were bought from Sigma Aldrich (St. Louis, MO, USA). The myeloperoxidase (MPO) (A044-1-1) assay kit was bought from Nanjing Jiancheng Bioengineering Institute (Nanjing, China). Tumor necrosis factor (TNF)-α (Cat# 430915) and interleukin (IL)-1β (Cat# 432615) enzyme-linked immunosorbent assay (ELISA) kits were obtained from Biolegend (San Diego, CA, USA). Specific antibodies including ZO-1 (1:1,000; #AF5145; RRID: AB_2837631), occludin (1:1,000; #DF7504; RRID: AB_2841004), claudin-3 (1:1,000; #AF0129; RRID: AB_2833313), Phosphorylation-p65 (p-p65, #AF2006; RRID: AB_2834435), p-65 (#AF5006; RRID: AB_2834847), p-IκB (#AF2002; RRID: AB_2834433), IκB (#AF5002; RRID: AB_2834792), p38(#AF6456; RRID: AB_2835277) and β-actin (1:1,000; #AF7018; RRID: AB_2839420) were purchased from Affinity Biosciences (OH, USA). NLRP3 (#15101), ASC (#67824), IL-1β (#12242), and p-p38 (#9211) were bought from Cell Signaling Technology (CST, Boston, USA).

### 2.17 Statistical analysis

GraphPad Prism 8.0 software was employed for statistical analysis. The data are presented as boxplots or mean ± SEM, and the representative data shown is one of three independent experiments. Kolmogorov–Smirnov test, D’Agostino’s and Pearson test, Shapiro–Wilk test and Anderson-Darling test were performed for normality and lognormality test. Significant differences were evaluated using the Mann–Whitney U test and one-way analysis of variance (ANOVA) followed by Tukey’s test. *p < 0.05 indicates a significant difference. Other specific statistical analyses are described in the relevant Methods section.

## 3 Results

### 3.1 Supplementation with YFF improved mastitis in SARA dairy goats

**Figure 1:**
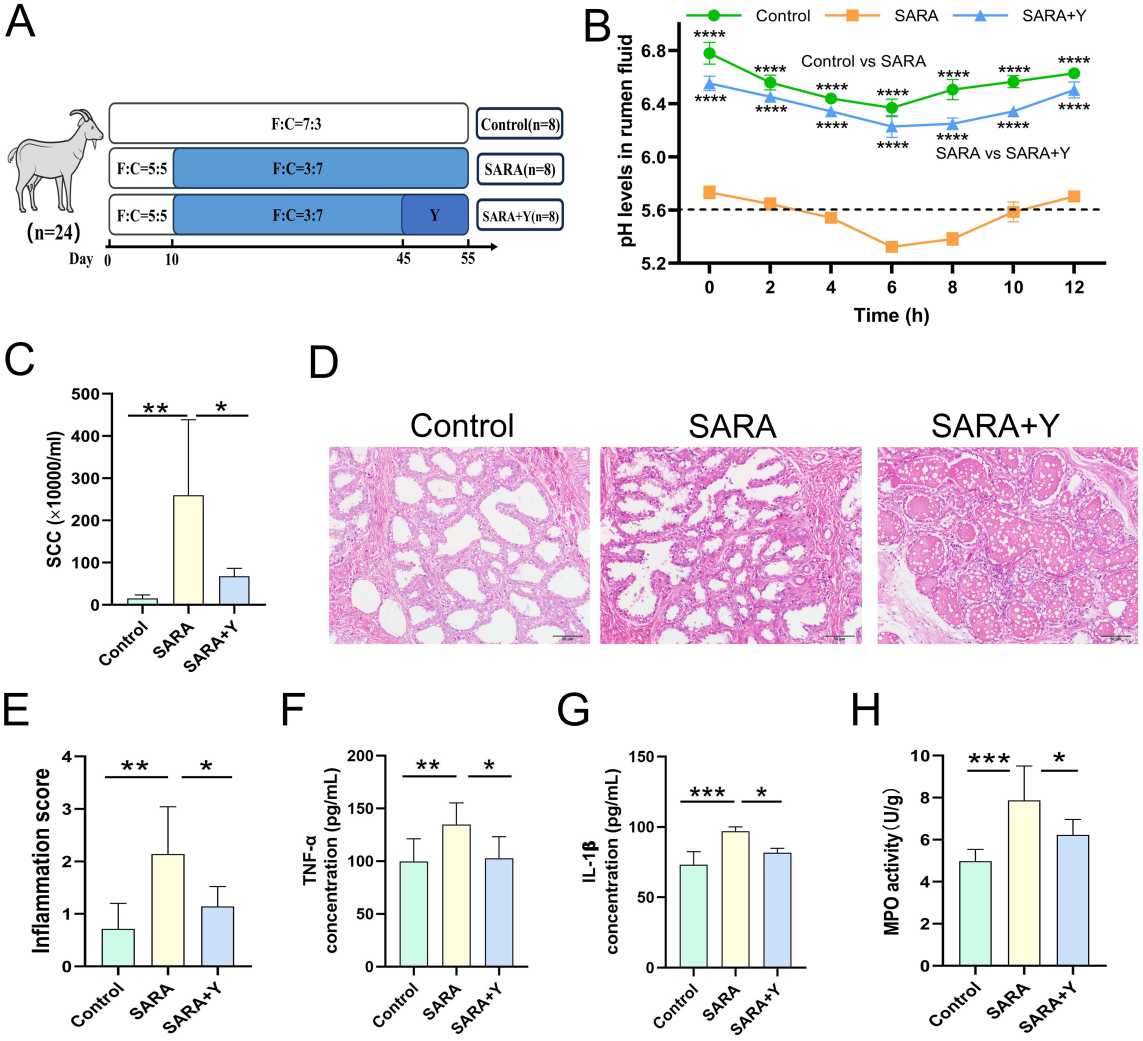
Supplementation with YFF improved mastitis in SARA dairy goats. Dairy goats were randomly divided into three groups. **(A)** Grouping and feeding methods of dairy goats. **(B)** On the first day after the end of the experiment, the pH of dairy goats was measured at different times during the day (*n* = 3). **(C)** SCC. **(D)** Histopathologic sections of mammary tissues (scale bar, 50 μm). **(E)** The inflammatory score based on the result of pathological damage was assessed (*n* = 6). **(F)** The expression of TNF-α in the mammary gland. **(G)** The expression of IL-1β in the mammary gland. **(H)** MPO activity of the mammary gland. Data were expressed as means ± SEM **(B,C,E–H)** and statistical significances were analyzed with ANOVA, followed by the Tukey test (**B,C,E–H**). *p < 0.05, **p < 0.01, ***p < 0.001, and ****p < 0.0001 indicate significance.

**Figure 2:**
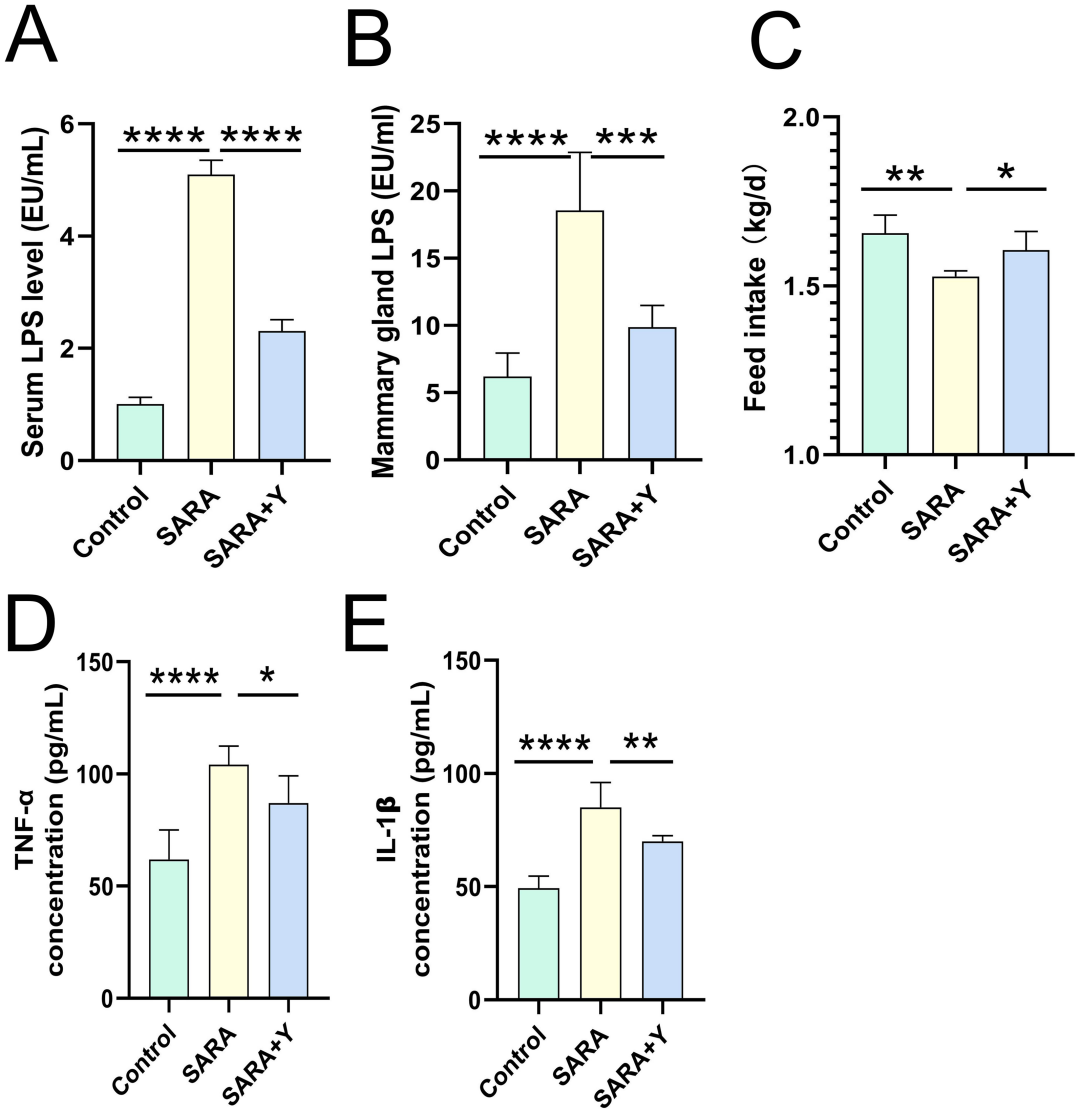
Supplementation with YFF improved systemic inflammation in SARA dairy goats. **(A)** Serum LPS levels. **(B)** Mammary gland LPS levels. **(C)** Feed intake. **(D)** The expression of TNF-α in the mammary gland. **(E)** The expression of IL-1β in the mammary gland. Data were expressed as means ± SEM **(A–E)** and statistical significances were analyzed with ANOVA, followed by the Tukey test **(A–E)**. *p < 0.05, **p < 0.01, ***p < 0.001, and ****p < 0.0001 indicate significance.

Different groups of dairy goats were fed roughage diets and HCD in different proportions (Figure 1A). On the day after the last day of feeding, the pH of rumen fluid was measured at different time points in different groups of dairy goats. The results indicated that, in contrast to the control group, long-term feeding of HCD led to a significant decrease in the pH of rumen fluid in dairy goats. The pH value was less than 5.6 for more than 3 h per day, which indicates the presence of SARA symptoms. The pH value of rumen fluid in dairy goats returned to normal after the supplementation of YFF (Figure 1B). Subsequently, SCC in the milk of dairy goats was detected. Compared with the control group, the SCC in the SARA group was significantly increased compared with that in the SARA group (Figure 1C). SCC is one of the criteria for the diagnosis of mastitis (Yuanhang et al., 2023), so this result suggests the occurrence of mastitis in the SARA group of dairy goats, and the supplementation of YFF may be helpful in the treatment of mastitis. Furthermore, to confirm the effects of SARA and YFF on dairy goat mastitis, we performed H&E staining of dairy goat mammary tissue (Figures 1D,E). The results showed that compared with the control group, the mammary tissue of goats in the SARA group showed destruction of acinar structure and infiltration of inflammatory cells, which were improved in the SARA+Y group. The levels of pro-inflammatory cytokines in the breast tissue were also examined, and the results showed that the levels of TNF-α (Figure 1F) and IL-1β (Figure 1G) were significantly increased in the SARA group compared with the control group, and then decreased after the supplementation of YFF. MPO activity also showed the same trend (Figure 1H). Evidence has shown that rumen-derived LPS induces mastitis through circulation into the mammary gland (Xiaoyu et al., 2022). Therefore, we examined the serum and mammary LPS levels in different groups of dairy goats. SARA significantly increased serum and mammary LPS levels compared with the control group, and this result was reversed by YFF supplementation (Figures 2A,B). In addition, several physiological parameters of dairy goats were examined. The results showed that SARA caused a decrease in feed intake in dairy goats, which was restored by the supplementation of YFF (Figure 2C). Blood biochemical indexes were measured in dairy goats under different treatments (Table 2). The results showed that serum globulin (GLOB) was significantly higher in SARA dairy goats than in control dairy goats. Total protein (TP) and the white globulins ratio (A/G) were higher in SARA dairy goats than in control goats, but there was no significant difference in albumin (ALB), which suggested systemic immune activation in SARA dairy goats. In addition, serum alanine aminotransferase (ALT) was significantly higher and alkaline phosphatase (ALP) was significantly lower in the SARA group than in the control group. Meanwhile, blood urea nitrogen (BUN) was significantly higher in SARA dairy goats than in control dairy goats. The blood urea nitrogen creatinine ratio (BUN/Cr), creatinine (Cr), and glucose (GLu) in the SARA group were higher than those in the control group. Corresponding to the above results, compared with the SARA group, the supplementation of YFF significantly reduced GLOB and BUN/Cr, significantly increased ALP, and decreased TP, A/G, ALT, Cr, BUN, and GLu in serum. Complete blood count was examined in dairy goats under different treatments (Table 3). Compared with the control group, white blood cell count (WBC), neutrophil count (Neu), and eosinophil count (Eos) in the SARA group were significantly increased. These changes were all improved after supplementation with YFF. Finally, serum expression of the proinflammatory cytokines TNF-*α* (Figure 2D) and IL-1β (Figure 2E) similarly increased after HCD feeding and decreased after YFF supplementation. These results suggest that long-term HCD induces SARA and subsequently mastitis in dairy goats, and that the symptoms of mastitis can be significantly improved by supplementation with YFF.

**Table 2:** Blood biochemical indexes.

Characteristics	Control	SARA	SARA+Y	*p*-value	
				Control vs. SARA	SARA vs. SARA+Y
ALB (g/L)	30.4 ± 3.54	31.35 ± 6.22	30.89 ± 4.31	0.7999	0.948
TP (g/L)	71.74 ± 12.73	80.25 ± 16.83	72.94 ± 13.93	0.0964	0.1693
GLOB (g/L)	41.34 ± 9.19	48.9 ± 10.61	42.05 ± 9.62	0.0199	0.0362
A/G	0.74 ± 0.07	0.64 ± 0.11	0.74 ± 0.06	0.0643	0.0743
ALT (U/L)	20.38 ± 11.31	25 ± 12.02	20.88 ± 5.66	0.1257	0.1857
Cr (umol/L)	71.23 ± 27.79	80.98 ± 35.21	71.61 ± 21.28	0.4063	0.4346
BUN (mmol/L)	5.90 ± 2.19	8.49 ± 3.48	6.38 ± 2.08	0.0373	0.0172
BUN/ Cr	83.86 ± 4.39	98.72 ± 3.67	95.13 ± 9.86	<0.0001	0.0018
GLu (mmol/L)	3.46 ± 1.65	3.76 ± 1.30	3.38 ± 1.02	0.5681	0.403

**Table 3:** Complete blood count.

Characteristics	Control	SARA	SARA+Y	*p*-value	
				Control vs. SARA	SARA vs. SARA+Y
WBC	9.747 ± 1.00	15.99 ± 4.32	12.06 ± 2.26	0.0409	0.2636
Neu%	52.3 ± 21.35	63.1 ± 32.53	50.84 ± 5.23	0.4571	0.4032
Lym%	39.95 ± 16.83	25.98 ± 30.26	43.08 ± 4.17	0.2639	0.1727
Mon%	6.32 ± 5.09	8.47 ± 0.35	4.56 ± 1.41	0.8098	0.5411
Eos%	1.35 ± 0.49	2.35 ± 1.91	1.44 ± 0.21	0.1140	0.1833
Bas%	0.08 ± 0.07	0.1 ± 0	0.08 ± 0.14	0.8930	0.8626
Neu#	5.07 ± 3.36	9.495 ± 4.77	6.03 ± 1.97	0.0147	0.0498
Lym#	3.83 ± 2.15	3.09 ± 0.93	5.32 ± 0.28	0.7794	0.1636
Mon#	0.65 ± 0.60	1.44 ± 0.45	0.54 ± 0.06	0.5524	0.4949
Eos#	0.12 ± 0.08	0.29 ± 0.02	0.17 ± 0.06	0.0019	0.0266
Bas#	0.02 ± 0.07	0.01 ± 0.01	0.006 ± 0.01	0.9741	0.8917
RBC	6.30 ± 0.55	5.59 ± 0.33	5.68 ± 0.33	0.3408	0.9847
HGB	81.5 ± 19.80	69.33 ± 14.85	76 ± 12.73	0.3255	0.7221
HCT	16.13 ± 2.12	13.97 ± 0.99	14.08 ± 0.28	0.2990	0.9987
MCV	25.57 ± 1.20	24.8 ± 0.28	24.78 ± 0.92	0.2302	0.9990
MCH	12.97 ± 1.98	12.35 ± 1.56	13.48 ± 3.04	0.8453	0.6063
MCHC	506 ± 55.15	496.6 ± 56.57	542.9 ± 103.2	0.9722	0.5488
RDW-CV	25.25 ± 2.83	23.53 ± 1.06	23.96 ± 3.04	0.4717	0.9564
RDW-SD	23.42 ± 3.32	21.33 ± 0.92	21.48 ± 3.89	0.4237	0.9959
PLT	2,280 ± 226.98	2,449 ± 93.34	2,661 ± 1,645	0.9558	0.9374
MPV	7.83 ± 0	7.68 ± 0.07	7.84 ± 0.28	0.8171	0.6423
PDW	4.2 ± 0.35	4.12 ± 0	4.26 ± 1.48	0.9719	0.9263
PCT	1.76 ± 0.17	1.90 ± 0.07	2.07 ± 1.20	0.9547	0.9260

### 3.2 The supplement of YFF improved the mastitis of dairy goats by improving the blood-milk barrier function, limiting the inflammatory response and reducing the level of oxidative stress

**Figure 3:**
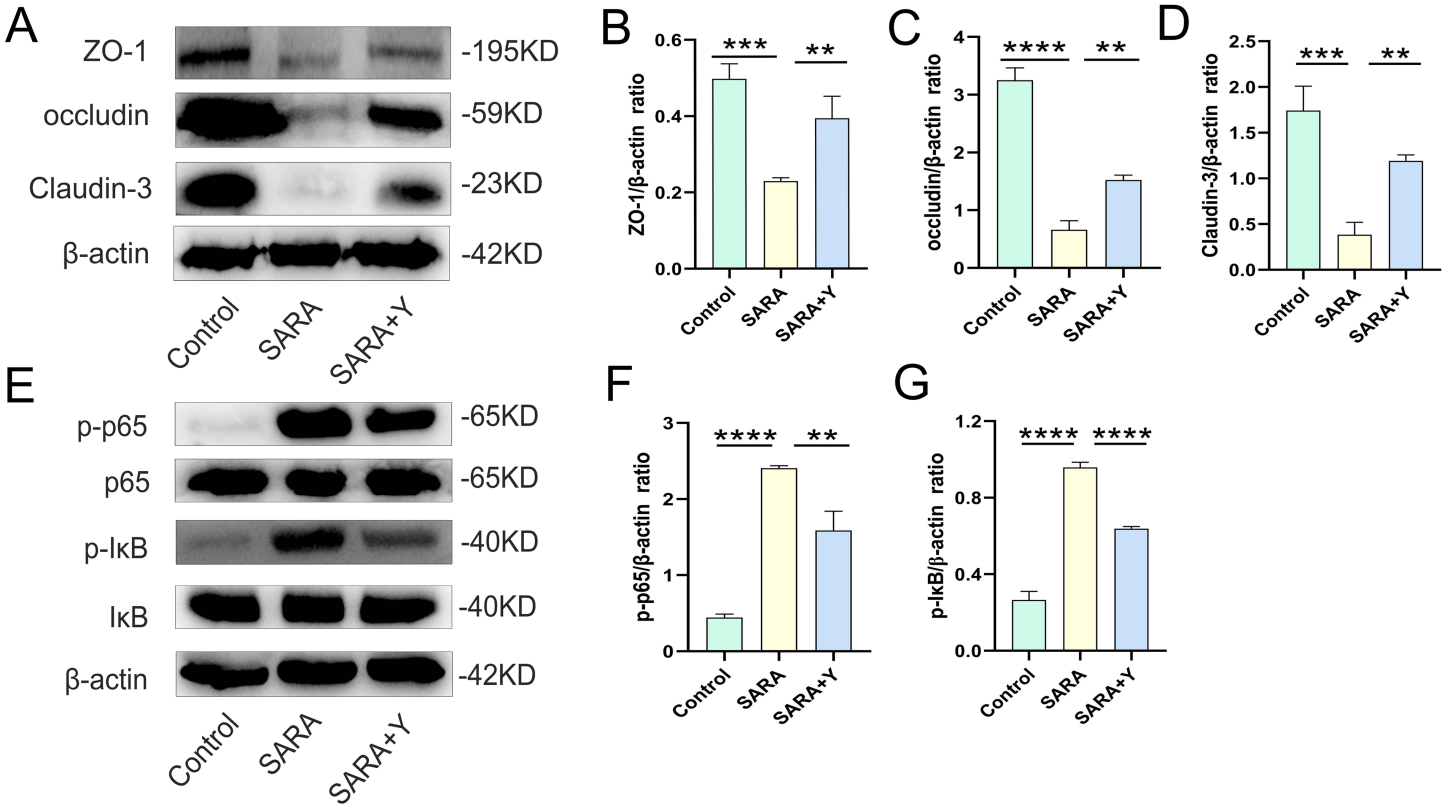
The supplement of YFF improved the mastitis of dairy goats by improving the blood-milk barrier function and inhibiting NF-κB inflammatory pathway activation. **(A)** The expressions of ZO-1, occludin and Claudin-3 in the mammary tissue were detected by western blotting. **(B–D)** Quantification of relative protein expression of ZO-1 **(B)**, occludin **(C)** and Claudin-3 **(D)** to β-actin. **(E)** The expressions of p65, p-p65, IκB and p-IκB in the mammary tissue were detected by western blotting. **(F,G)** Quantification of relative protein expression of p-p65 **(F)** and p-IκB **(G)** to β-actin. Data were expressed as means ± SEM **(B–D,F,G)** and statistical significances were analyzed with ANOVA, followed by the Tukey test **(B–D,F,G)**. *p < 0.05, **p < 0.01, ***p < 0.001, and ****p < 0.0001 indicate significance.

**Figure 4:**
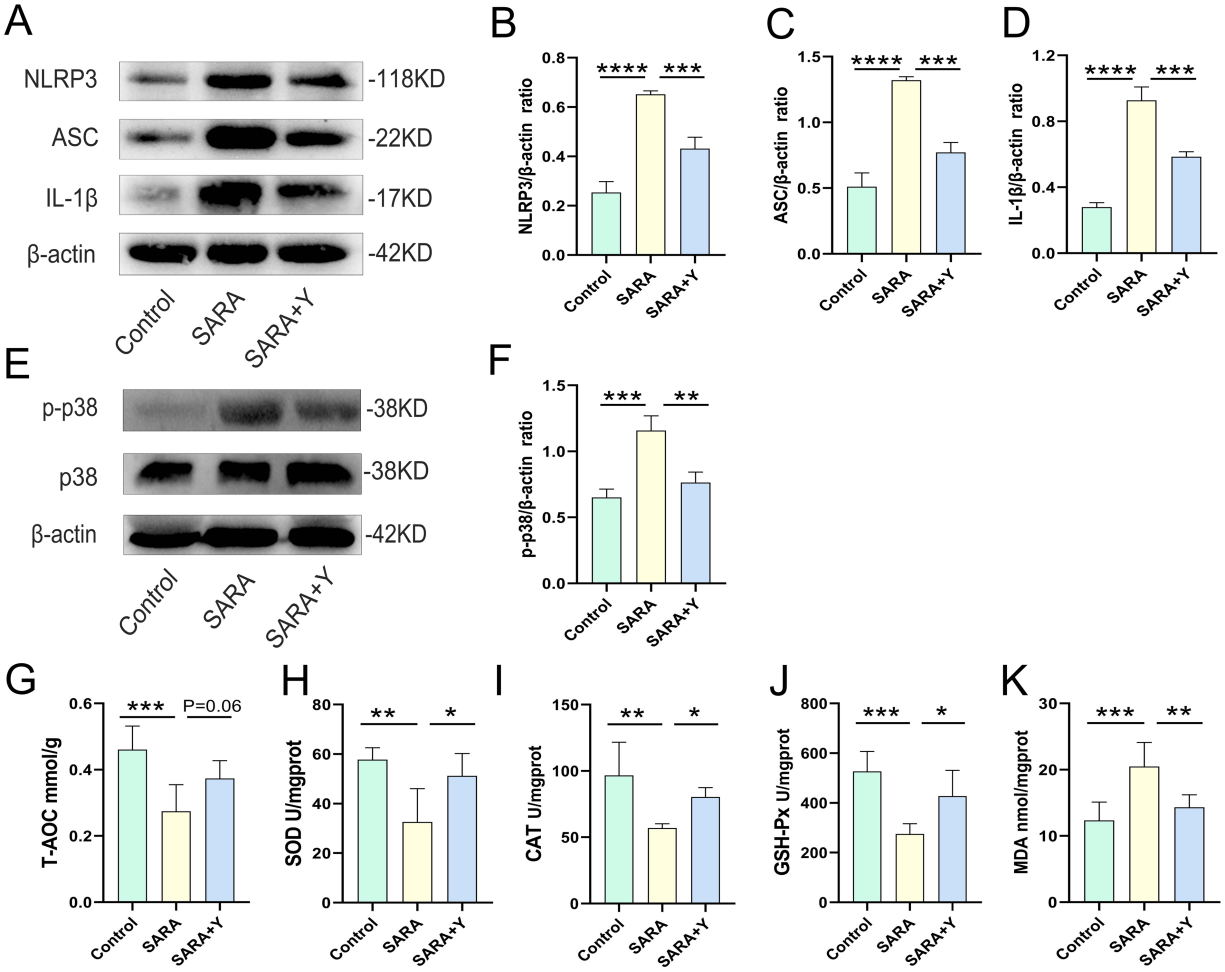
The supplement of YFF improved the mastitis of dairy goats by inhibiting NLRP3 and MAPK inflammatory pathway activation and reducing the level of oxidative stress. **(A)** The expressions of NLRP3, ASC and IL-1β in the mammary tissue were detected by western blotting. **(B–D)** Quantification of relative protein expression of NLRP3 **(B)**, ASC **(C)** and IL-1β **(D)** to β-actin. **(E)** The expressions of p38, and p-p38 in the mammary tissue were detected by western blotting. **(F)** Quantification of relative protein expression of p-p38 to β-actin. **(G)** The expression of T-AOC in the mammary gland. **(H)** The expression of SOD in the mammary gland. **(I)** The expression of CAT in the mammary gland. **(J)** The expression of GSH-Px in the mammary gland. **(K)** The expression of MDA in the mammary gland. Data were expressed as means ± SEM **(B–D,F–K)** and statistical significances were analyzed with ANOVA, followed by the Tukey test **(B–D,F–K)**. *p < 0.05, **p < 0.01, ***p < 0.001, and ****p < 0.0001 indicate significance.

To determine the specific mechanism by which YFF alleviates mastitis in dairy goats, we conducted follow-up experiments. The blood-milk barrier is a specific structure that functions as a barrier to prevent foreign substances from entering the mammary gland from either the blood or the external environment (Xiaoyu et al., 2020). WB was uesd to examine the expression of TJ proteins in the mammary glands of dairy goats from different treatment groups (Figure 3A). The results indicated that the levels of TJ proteins ZO-1 (Figure 3B), occludin (Figure 3C), and Claudin-3 (Figure 3D) were significantly decreased in the SARA group compared to the control group, but were restored in the SARA+Y group. This suggests that when SARA occurs, the blood-milk barrier is disrupted, and the integrity of the blood-milk barrier can be significantly enhanced by supplementing with YFF. Subsequently, WB was used to examine the activation of multiple inflammatory pathways in order to determine the role of YFF in the inflammatory response. Firstly, the phosphorylation of p65 and IκB was significantly increased in the SARA group compared to the control group, however, this result was reversed by the supplementation of YFF (Figures 3E–G). Secondly, compared with the control group, the expression of NLRP3, ASC, and IL-1β was significantly increased in the SARA group and then decreased in the SARA+Y group (Figures 4A–D). Finally, the phosphorylation level of p38, which was significantly increased in the SARA group compared to the control group, was also decreased in the SARA+Y group (Figures 4E–F). These results demonstrated that the inflammatory signaling pathways NF-κB, NLRP3, and MAPK were activated in the SARA group, and the activation of these pathways was significantly inhibited by the supplementation of YFF. Dairy goats and cows are often plagued by oxidative stress during lactation (Song et al., 2021). Therefore, we examined the level of oxidative stress in the mammary gland. The results showed that the levels of total antioxidant capacity (T-AOC) (Figure 4G), superoxide dismutase (SOD) (Figure 4H), catalase (CAT) (Figure 4I), and glutathione peroxidase (GSH-Px) (Figure 4J) in the SARA group were significantly lower than those in the control group, and the level of malondialdehyde (MDA) (Figure 4K) was significantly higher than that in the control group. On the contrary, these changes were reversed in the SARA+Y group. This indicates that the occurrence of SARA leads to an increase in the level of oxidative stress in mammary tissue, and the supplementation of YFF can significantly improve oxidative stress. In conclusion, supplementing with YFF can improve the symptoms of mastitis by enhancing blood-milk barrier function, limiting the inflammatory response, and reducing the level of oxidative stress.

### 3.3 YFF supplementation improves rumen function in dairy goats

**Figure 5:**
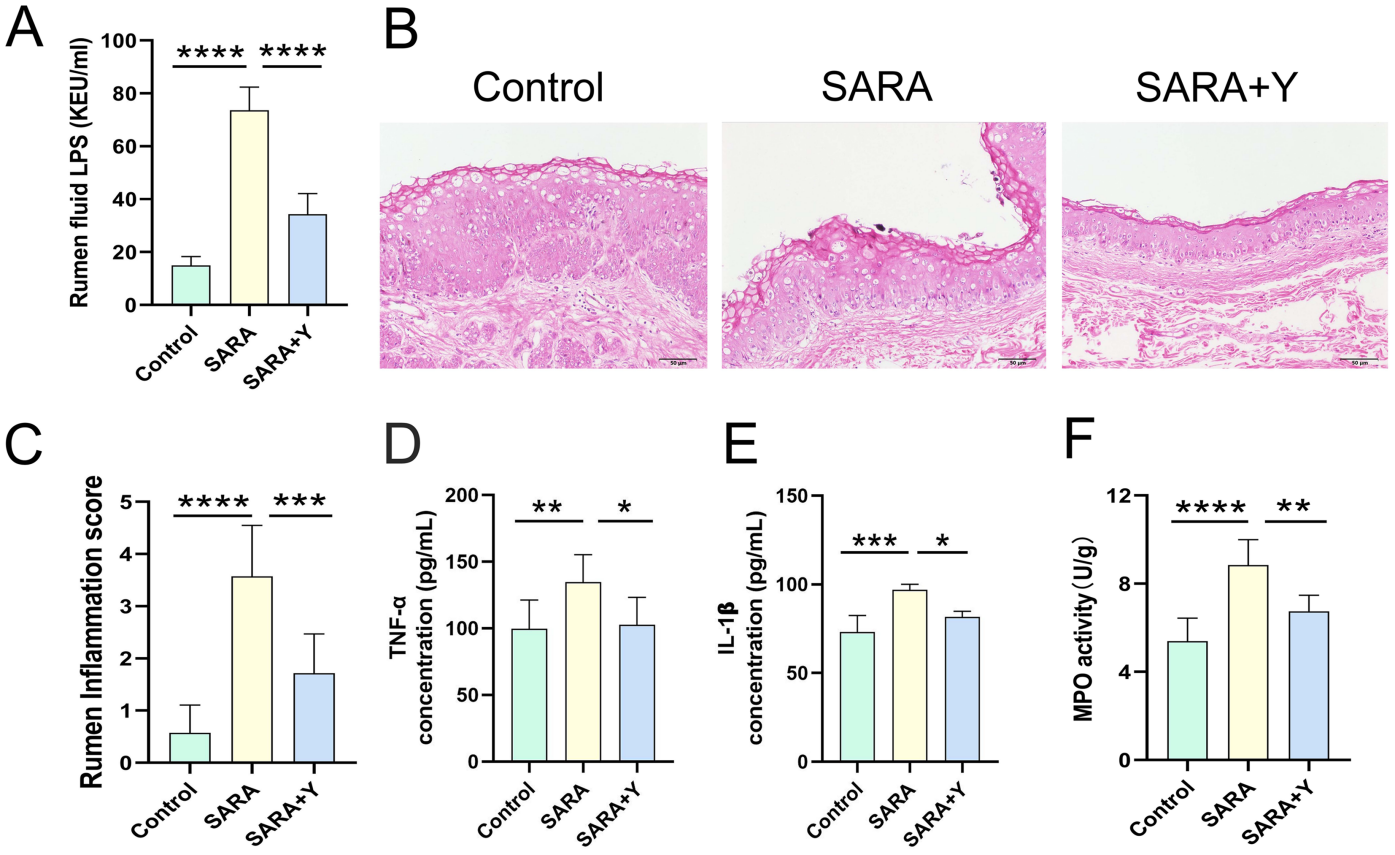
YFF supplementation improves rumen function in dairy goats. **(A)** Rumen LPS levels. **(B)** Histopathologic sections of rumen tissues (scale bar, 50 μm). **(C)** The inflammatory score based on the result of pathological damage was assessed (*n* = 6). **(D)** The expression of TNF-α in the rumen gland **(E)** The expression of IL-1β in the rumen gland. **(F)** MPO activity of the rumen gland. Data were expressed as means ± SEM **(A,C–F)** and statistical significances were analyzed with ANOVA, followed by the Tukey test **(A,C–F)**. *p < 0.05, **p < 0.01, ***p < 0.001, and ****p < 0.0001 indicate significance.

**Figure 6:**
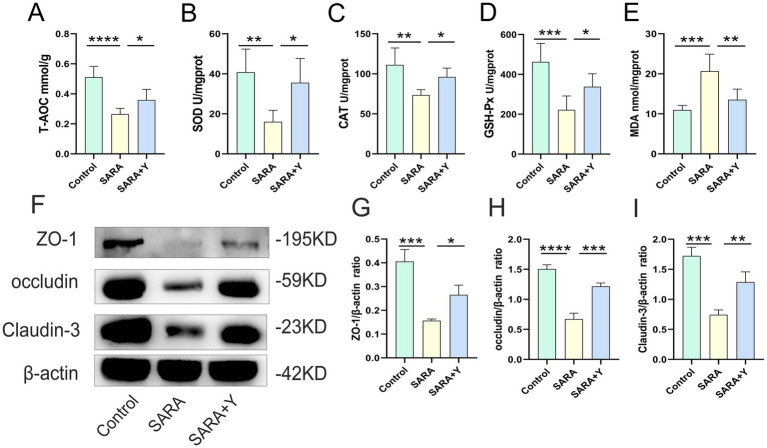
YFF supplementation reduces the level of oxidative stress and improves rumen barrier function in dairy goats. **(A)** The expression of T-AOC in the mammary gland. **(B)** The expression of SOD in the mammary gland. **(C)** The expression of CAT in the mammary gland. **(D)** The expression of GSH-Px in the mammary gland. **(E)** The expression of MDA in the mammary gland. **(F)** The expressions of ZO-1, occludin and Claudin-3 in the mammary tissue were detected by western blotting. **(L–N)** Quantification of relative protein expression of ZO-1 **(G)**, occludin **(H)** and Claudin-3 **(I)** to β-actin. Data were expressed as means ± SEM **(A–E,G–I)** and statistical significances were analyzed with ANOVA, followed by the Tukey test **(A–E,G–I)**. *p < 0.05, **p < 0.01, ***p < 0.001, and ****p < 0.0001 indicate significance.

To further clarify the therapeutic effect of YFF on SARA-induced mastitis, we examined the function of the rumen in dairy goats. Firstly, the content of LPS in the rumen fluid was significantly elevated (Figure 5A). H&E staining demonstrated that, in comparison with the control group, the ruminal epithelium of dairy goats in the SARA group exhibited obvious damage, low integrity, hyperplasia, and immune cell infiltration (Figures 5B,C). Additionally, the levels of ruminal pro-inflammatory cytokines (Figures 5D,E) and MPO activity (Figure 5F) were significantly increased in the SARA group. Consistently, the level of oxidative stress in the rumen was also markedly increased in the SARA group (Figures 6A–E). Moreover, the detection of TJ proteins in the rumen barrier revealed that the protein expression levels of ZO-1, occludin, and Claudin-3 were all significantly reduced in the SARA group compared to the control group (Figures 6F–I). On the contrary, after feeding with YFF, the content of LPS was significantly decreased (Figure 5A). The pathological changes of rumen tissues were significantly improved (Figures 5B,C), and the levels of pro-inflammatory cytokines (Figures 5D,E), MPO activity (Figure 5F), and oxidative stress level (Figures 6A–E) were significantly decreased. TJ protein expressions were significantly increased (Figures 6F–I) in the rumen. These results indicate that the permeability of the rumen of SARA dairy goats is significantly increased, which leads to the passage of LPS through the rumen barrier into the blood circulation and the occurrence of mastitis. At the same time, significant inflammatory changes occur in the rumen. However, the supplementation of YFF can significantly improve the function of the rumen barrier and reduce the level of ruminal inflammation.

### 3.4 YFF supplementation improves rumen microbiota in dairy goats

**Figure 7:**
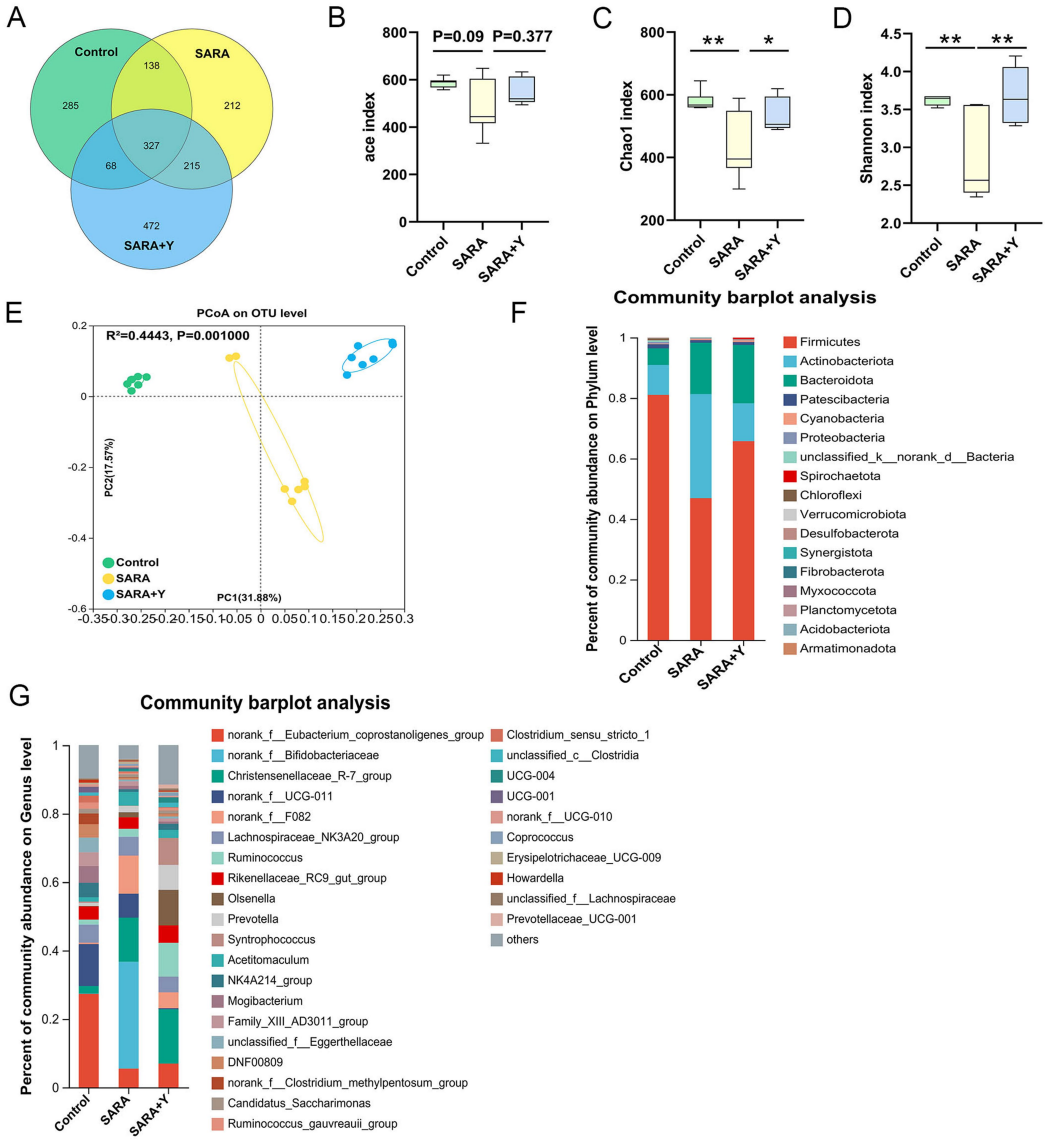
YFF supplementation improves rumen microbiota in dairy goats. 16S rRNA sequencing was used to analyze the rumen microbiota. **(A)** The Venn diagram shows the proportion of differential bacteria in the rumen bacterial microbiota of the control, SARA, and SARA+Y groups. **(B–D)** Alpha diversity analysis from the indicated groups (*n* = 6), ace index **(B)**, Chao1 index **(C)**, and Shannon index **(D)**. **(E)** PCoA showed different rumen microbial structure between the control, SARA, and SARA+Y groups based on unweighted UniFrac distance (*R*^2^ = 0.4443, *p* = 0.001, *n* = 6). **(F)** Bacterial composition at the phylum level in the indicated groups (*n* = 6). **(G)** Bacterial composition at the genus level in the indicated groups (*n* = 6). Data were expressed as means ± SEM **(B–D)** and statistical significances were analyzed with ANOVA, followed by the Tukey test **(B–D)**. *p < 0.05, and **p < 0.01 indicate a significant difference.

**Figure 8:**
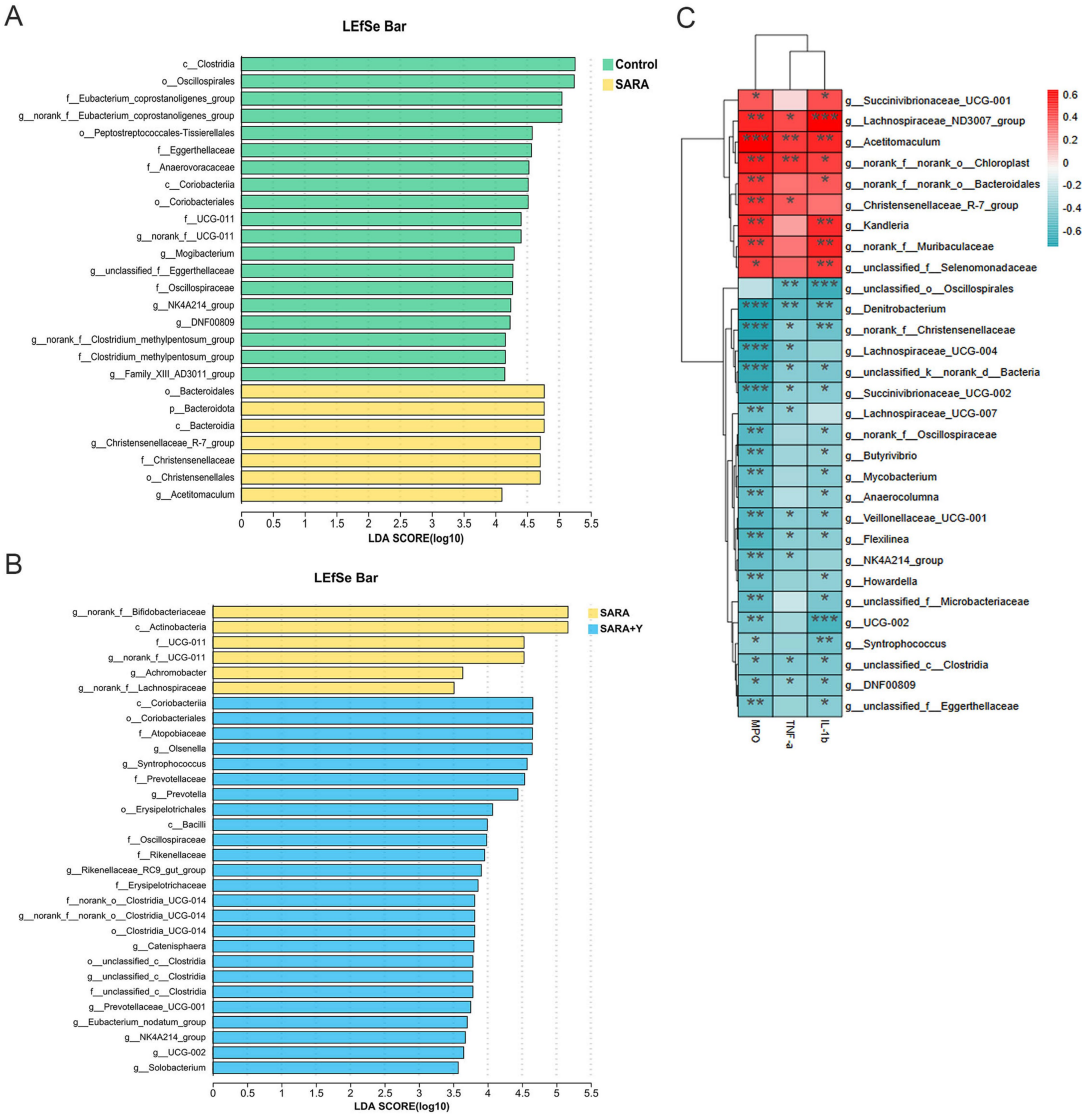
LEfSe analysis of rumen microbiome and correlation analysis of inflammatory markers in different treatment groups. **(A)** LEfSe showed different bacterial taxa that were enriched in control and SARA groups (log10 LDA score > 3.5). **(B)** LEfSe showed different bacterial taxa that were enriched in SARA and SARA+Y groups (log10 LDA score > 3.5). **(C)** Spearman-parametric rank correlation matrix between inflammatory markers and microbiota relative abundance. The blue color represents a negative correlation, the red color represents a positive correlation. Spearman correlations between bacterial and biological parameters at corresponding were analyzed. Data were expressed as means ± SEM **(C)** and statistical significances were analyzed with ANOVA, followed by the Tukey test **(C)**. *p < 0.05, and **p < 0.01 indicate a significant difference.

16S rRNA sequencing was carried out on the rumen fluid of dairy goats in the control, SARA, and SARA+Y groups. Venn diagram analysis revealed that there were 465 bacterial species common to the rumen microbiota of dairy goats in the control group and the SARA group. Additionally, 353 bacterial species were unique to the rumen of dairy goats in the control group, and 427 bacterial species were unique to the rumen of dairy goats in the SARA group. In the rumen microbiota of dairy goats in the SARA group and the SARA+Y group, there were 542 bacterial species. Specifically, 350 bacterial species were unique to the rumen of dairy goats in the SARA group, and 540 bacterial species were unique to the rumen of dairy goats in the SARA+Y group (Figure 7A). Alpha diversity analysis indicated that the ace index (Figure 7B), Chao1 index (Figure 7C), and Shannon index (Figure 7D) of the rumen microbiota in the SARA group were lower than those in the control group. There were significant differences in the Chao1 index and Shannon index between the two groups. Moreover, these indexes were all increased after the supplementation of YFF. PCoA demonstrated that there were significant differences in the rumen microbiota structure of dairy goats in different treatment groups (Figure 7E). At the phylum level (Figure 7F), SARA decreased the abundance of Firmicutes and increased the abundance of Actinobacteriota and Bacteroidota. The supplementation of YFF restored the abundance of Firmicutes and Actinobacteria. At the genus level (Figure 7G), SARA increased the levels of *norank_f__Bifidobacteriaceae*, *norank_f__F082*, *Acetitomaculum*, and other bacteria. Meanwhile, the abundance of *norank_f__Eubacterium_coprostanoligenes_group*, *norank_f__UCG-011A*, and other bacteria was reduced. The supplementation of YFF increased the abundance of *Ruminococcus*, *Olsenella*, and other bacteria while decreasing the abundance of *norank_f__Bifidobacteriaceae*, *norank_f__F082*, *Acetitomaculum*, and other bacteria. The rumen microbiota composition was analyzed by LEfSe (Figures 8A,B). In the comparison between the control and SARA groups (Figure 8A), 19 bacteria, including *c__Clostridia*, were highly expressed in the control group, and 7 bacteria, including *o__Bacteridales*, were highly expressed in the SARA group. In the comparison between the SARA and SARA+Y groups (Figure 8B), six species of bacteria, including *g__norank_f__Bifidobacteriaceae*, were highly expressed in the SARA group. Twenty-five bacteria, including *o__Coriobacteriia*, were highly expressed in the SARA+Y group. Finally, by analyzing the correlation between microbiota and inflammatory markers (Figure 8C), we found that 21 bacterial genera such as *g__unclassified_o__Oscillospirales* and *g__Denitrobacterium* showed a significant negative correlation with TNF-α, IL-1β and MPO, which suggested that these bacterial genera may play a positive role in alleviating mastitis. On the contrary, nine genera such as *g__Lachnospiraceae_ND3007_group* and *g__Acetitomaculum* were positively correlated with these inflammatory markers, and these genera may be important causes of mastitis.

## 4 Discussion

Mastitis, characterized by complex etiology, high incidence rate, and difficult prevention and treatment, has always been one of the major diseases plaguing the healthy development of the global dairy industry (Xiaoyu et al., 2023). This disease not only leads to a reduction in milk production and quality but also poses a great threat to food safety and human health (Yue et al., 2024). Therefore, in order to maximize milk production, it is often necessary to increase the proportion of concentrate in the feed (Qu et al., 2021), which in turn increases the risk of SARA. SARA, as a common nutritional metabolic disease in ruminants, is mainly manifested by a decrease in rumen pH (Darío et al., 2020), accompanied by disturbances in the rumen microbiota (Tao et al., 2022), thereby increasing the chance of mastitis (Caijun et al., 2023b). Our study indicates that long-term feeding of HCD causes SARA in dairy goats. This leads to the destruction of the rumen barrier and the disorder of the rumen microbiota. In turn, this destroys the blood-milk barrier function, activates the inflammatory signaling pathway, induces oxidative stress, and finally results in the occurrence of mastitis in dairy goats. Supplementation of YFF on this basis can significantly alleviate the negative impacts caused by SARA and treat mastitis.

Microbial fermented feed has been widely employed in production practice due to its numerous advantages. Specifically, microbial fermentation of feed can enhance feed nutritional value, improve palatability, reduce anti-nutritional factors, and increase digestibility (Ting et al., 2024). Additionally, it is also of great significance in improving the balance of gastrointestinal microbiota (Siyu et al., 2022). Yeast, a facultative anaerobic fungus that is easy to survive and has a wide distribution, is often utilized in the food field as a starter culture and for adding nutritional value (Ahasanul et al., 2020). In the medical field, it is highly regarded because of its functions in regulating gut microbiota and enhancing immunity (Gu et al., 2022). Yeasts can also stimulate the growth and differentiation of intestinal epithelial cells and enhance the barrier function of the gut mucosa (Runqi et al., 2023). Some beneficial metabolites produced by yeasts during fermentation in the intestine, such as glucans and mannan, also have various beneficial functions (Hongli et al., 2020). Glucans, as a polysaccharide substance present in the cell wall of yeast, have a variety of beneficial functions, including activating immune cells (Hongli et al., 2020), promoting antibody production (Qian et al., 2023), facilitating the growth of beneficial bacteria (Pedro et al., 2023), exhibiting anti-tumor effects (Jingjing et al., 2009) and having antioxidant properties (Miloš et al., 2019). Mannan can also play a role in regulating intestinal function (Zhi-Yuan et al., 2022) and immune response (Arsenault et al., 2017). Yeast cultures play a positive role in microbial abundance and immune function in the context of a high-grain diet. Therefore, the use of yeast to ferment feed has broad development prospects (Pei and Lizhi, 2024).

SCC can reflect the health status of the breast and is an important parameter for the diagnosis of mastitis. Our study found that long-term feeding of HCD led to an increase in SCC. This was accompanied by breast tissue damage and a significant elevation in the levels of the proinflammatory cytokines TNF-α and IL-1β, suggesting the occurrence of mastitis. Simultaneously, the rumen pH decreased, and when the rumen pH was less than 5.6 for more than 3 h per day, it indicated the occurrence of SARA. Once SARA occurs, the low pH will cause the death of Gram-negative bacteria in the rumen and release large amounts of LPS (Chenxu et al., 2018). This LPS will cross the damaged rumen barrier and enter the bloodstream, leading to systemic low-grade inflammation in the body (Stefanska et al., 2017). This is consistent with the results of the present study. In our study, the SARA group was found to have increased ruminal LPS content, impaired ruminal epithelium, increased serum LPS content, impaired blood-milk barrier function, and increased mammary LPS content. Furthermore, the inflammatory signaling pathways NF-κB, NLRP3, and MAPK were activated in the mammary gland of the SARA group, resulting in a strong inflammatory response. At the same time, severe oxidative stress occurred in the mammary gland and rumen. Studies have shown that yeast hydrolysate could attenuate the inflammatory response and intestinal barrier injury in weaned piglets challenged with LPS (Runqi et al., 2023). Consistent with these findings, dietary supplementation with YFF significantly restored the integrity of the rumen epithelium and the function of the rumen barrier. Meanwhile, the function of the mammary blood-milk barrier was also improved, and the activation of inflammatory signaling pathways in the mammary gland was reduced. Yeast has also been shown to relieve oxidative stress (Lei et al., 2020). This was similarly confirmed by our results, which showed that supplementation with YFF alleviated oxidative stress in the rumen and mammary tissues of SARA dairy goats. These results fully demonstrate the role of YFF in the treatment of SARA-induced mastitis in dairy goats.

The gut microbiota has been demonstrated to be closely related to the function and occurrence of diseases in various distal organs. For instance, disruption of the gut microbiota can lead to neurodevelopmental disorders (Qinwen et al., 2023) and sleep disorders (Zhe et al., 2022). Beneficial microorganisms colonize in the intestine, regulate the balance of the gut microbiome, and further promote gut health. This healthy gut environment lays a solid foundation for the overall health of animals and plays a crucial role in the improvement of diseases. Our previous studies have also proven that the gut microbiota is closely related to the occurrence of mastitis (Xiaoyu et al., 2020). As the largest digestive and fermentation organ of ruminants, the ruminal microbiota can better represent the gut microbiota of ruminants. In our study, we found that during the process of inducing SARA in dairy goats through high-concentrate feeding, the diversity and richness of the rumen microbiota were decreased, and the structure of the rumen microbiota was significantly different from that of healthy dairy goats. At the phylum level, SARA causes a decrease in the abundance of *Firmicutes* and an increase in the abundance of Actinobacteriota and Bacteroidota. This change often indicates a risk of developing disease. A decrease in the abundance of Firmicutes was observed in the gut microbiota of depressed mice (Tian et al., 2022). An increased abundance of Bacteroidota occurs in patients with irritable bowel syndrome (Pittayanon et al., 2019). The reduction of beneficial bacteria and the expansion of pathogenic bacteria increase the risk of diseases for the organism. This is consistent with our findings that SARA-induced changes in the rumen microbiota led to systemic pathological changes including mastitis. Yeasts and their metabolites play a significant role in regulating the gut microbiota (Gu et al., 2022). At the phylum level, supplementation with YFF restores the abundance of Firmicutes and Actinobacteria. At the genus level, supplementation with YFF increases the abundance of *Ruminococcus* and *Olsenella*. The enrichment of *Ruminococcus* can alleviate metabolic disorders such as metabolic dysfunction-associated steatotic liver disease (Zhang et al., 2024). The enrichment of the genus *Olsenella* is also strongly associated with maintaining colonic barrier integrity and reducing obesity-related gene expression and metabolic changes (Li et al., 2021). These results fully illustrate the important role played by YFF in the regulation of the rumen microbiota. It can improve the health and productivity of animals, enhance their resistance to various diseases, and ensure the safety and high quality of dairy products. This study creatively proposed the use of microbial fermentation feed to alleviate mastitis in dairy goats, which is helpful to improve the health level of ruminants, balance the relationship between production and health, and provide a basis for the development of microecological feed additives.

## 5 Conclusion

In conclusion, dietary supplementation with YFF can treat mastitis in dairy goats caused by long-term HCD. This is accomplished by restoring blood-milk barrier function, inhibiting the inflammatory response, and reducing oxidative stress. Additionally, it enhances the rumen barrier function, reduces oxidative stress, and regulates the disorder of rumen microbiota. With people’s continuous attention to food safety and animal welfare, the application scope of YFF will surely become more extensive. In the future, we can further deeply explore the action mechanism of YFF and optimize the production process to provide stronger support for the sustainable development of animal husbandry and the dairy industry.

### 5.1 Limitations of the study

Although our study clearly identified the therapeutic effect of YFF on HCD induced mastitis in dairy goats, and confirmed the effects of YFF on improving blood-milk barrier function, inhibiting inflammatory response and oxidative stress, and regulating gut microbiome, we did not elucidate the specific mechanism of action, which is also the limitation of this study. The active components of YFF are diverse, and the regulatory functions of β-glucan and mannan on host innate immunity may be the focus of future research. We also look forward to clarifying the main molecular mechanism of YFF in detail in the future.

## Data Availability

The datasets presented in this study can be found in online repositories. The names of the repository/repositories and accession number(s) can be found in the article/supplementary material.

## References

[ref1] Adak A. Khan M. (2019). An insight into gut microbiota and its functionalities. Cell. Mol. Life Sci. CMLS 76, 473–493. doi: 10.1007/s00018-018-2943-4, PMID: 30317530 PMC11105460

[ref2] Agus A. Clément K. Sokol H. (2021). Gut microbiota-derived metabolites as central regulators in metabolic disorders. Gut 70, 1174–1182. doi: 10.1136/gutjnl-2020-323071, PMID: 33272977 PMC8108286

[ref3] Ahasanul K. Natela G. Mohammed A. (2020). Kluyveromyces marxianus: an emerging yeast cell factory for applications in food and biotechnology. Int. J. Food Microbiol. 333:108818. doi: 10.1016/j.ijfoodmicro.2020.108818 32805574

[ref4] Ali Assar S. Zhiwei L. Chen Q. Juanzi W. Nighat S. Xiaoxian Z. (2019). Potential effect of the microbial fermented feed utilization on physicochemical traits, antioxidant enzyme and trace mineral analysis in rabbit meat. J. Anim. Physiol. Anim. Nutr. (Berl.) 104, 767–775. doi: 10.1111/jpn.13252, PMID: 31742797

[ref5] Arsenault R. Lee J. Latham R. Carter B. Kogut M. (2017). Changes in immune and metabolic gut response in broilers fed β-mannanase in β-mannan-containing diets. Poult. Sci. 96, 4307–4316. doi: 10.3382/ps/pex246, PMID: 29053819

[ref6] Caijun Z. Lijuan B. Yihong Z. Keyi W. Min Q. Lianjun F.. (2023a). A fiber-enriched diet alleviates *Staphylococcus aureus*-induced mastitis by activating the HDAC3-mediated antimicrobial program in macrophages via butyrate production in mice. PLoS Pathog. 19:e1011108. doi: 10.1371/journal.ppat.1011108, PMID: 36656870 PMC9888710

[ref7] Caijun Z. Xiaoyu H. Lijuan B. Keyi W. Lianjun F. Min Q.. (2021). Aryl hydrocarbon receptor activation by *Lactobacillus reuteri* tryptophan metabolism alleviates *Escherichia coli*-induced mastitis in mice. PLoS Pathog. 17:e1009774. doi: 10.1371/journal.ppat.1009774, PMID: 34297785 PMC8336809

[ref8] Caijun Z. Xiaoyu H. Min Q. Lijuan B. Keyi W. Xiangyue M.. (2023b). Sialic acid exacerbates gut dysbiosis-associated mastitis through the microbiota-gut-mammary axis by fueling gut microbiota disruption. Microbiome 11:78. doi: 10.1186/s40168-023-01528-8, PMID: 37069691 PMC10107595

[ref9] Chenxu Z. Yazhou W. Zhicheng P. Xudong S. Guoquan S. Xue Y.. (2018). Subacute ruminal acidosis suppressed the expression of MCT1 in rumen of cows. J. Cell. Physiol. 234, 11734–11745. doi: 10.1002/jcp.27829 30536938

[ref10] Dai H. Liu X. Yan J. Aabdin Z. Bilal M. Shen X. (2017). Sodium butyrate ameliorates high-concentrate diet-induced inflammation in the rumen epithelium of dairy goats. J. Agric. Food Chem. 65, 596–604. doi: 10.1021/acs.jafc.6b04447, PMID: 28032994

[ref11] Darío V.-T. Julián R.-V. John V. Juan M.-E. Juan A.-M. (2020). Incidence and effects of subacute ruminal acidosis and subclinical ketosis with respect to postpartum anestrus in grazing dairy cows. Heliyon 6:e03712. doi: 10.1016/j.heliyon.2020.e03712, PMID: 32274437 PMC7132069

[ref12] Elmhadi M. E. Ali D. K. Khogali M. K. Wang H. (2022). Subacute ruminal acidosis in dairy herds: microbiological and nutritional causes, consequences, and prevention strategies. Anim Nutr 10, 148–155. doi: 10.1016/j.aninu.2021.12.008, PMID: 35702144 PMC9168481

[ref13] Fu Y. He Y. Xiang K. Zhao C. He Z. Qiu M.. (2022). The role of rumen microbiota and its metabolites in subacute ruminal acidosis (SARA)-induced inflammatory diseases of ruminants. Microorganisms 10:1495. doi: 10.3390/microorganisms10081495, PMID: 35893553 PMC9332062

[ref14] Fung T. Olson C. Hsiao E. (2017). Interactions between the microbiota, immune and nervous systems in health and disease. Nat. Neurosci. 20, 145–155. doi: 10.1038/nn.4476, PMID: 28092661 PMC6960010

[ref15] Garcia S. N. Mpatswenumugabo J. P. M. Ntampaka P. Nandi S. Cullor J. S. (2023). A one health framework to advance food safety and security: an on-farm case study in the Rwandan dairy sector. One Health 16:100531. doi: 10.1016/j.onehlt.2023.100531 37363252 PMC10288054

[ref16] Gozho G. N. Krause D. O. Plaizier J. C. (2007). Ruminal lipopolysaccharide concentration and inflammatory response during grain-induced subacute ruminal acidosis in dairy cows. J. Dairy Sci. 90, 856–866. doi: 10.3168/jds.S0022-0302(07)71569-2, PMID: 17235162

[ref17] Gu Y. Wang C. Qin X. Zhou B. Liu X. Liu T.. (2022). Saccharomyces boulardii, a yeast probiotic, inhibits gut motility through upregulating intestinal serotonin transporter and modulating gut microbiota. Pharmacol. Res. 181:106291. doi: 10.1016/j.phrs.2022.106291, PMID: 35690329

[ref18] Guetterman H. Huey S. Knight R. Fox A. Mehta S. Finkelstein J. (2022). Vitamin B-12 and the gastrointestinal microbiome: a systematic review. Adv. Nutr. (Bethesda, Md.). 13, 530–558. doi: 10.1093/advances/nmab123, PMID: 34612492 PMC8970816

[ref19] Guo W. Guo X. Xu L. Shao L. Zhu B. Liu H.. (2022). Effect of whole-plant corn silage treated with lignocellulose-degrading bacteria on growth performance, rumen fermentation, and rumen microflora in sheep. Animal 16:100576. doi: 10.1016/j.animal.2022.100576, PMID: 35777297

[ref20] Hans Ulrich S. Thomas H. Gerd R. B. (2020). The etiology of rheumatoid arthritis. J. Autoimmun. 110:102400. doi: 10.1016/j.jaut.2019.102400 31980337

[ref21] Hongli S. Yinghua Y. Danhong L. Peng Z. Peng Z. Minmin H.. (2020). Β-glucan attenuates cognitive impairment via the gut-brain axis in diet-induced obese mice. Microbiome 8:143. doi: 10.1186/s40168-020-00920-y, PMID: 33008466 PMC7532656

[ref22] Huizhi Y. Sufang H. Shufei Z. Yuling X. Yaoguang Z. Han L.. (2022). Microbial properties of raw milk throughout the year and their relationships to quality parameters. Food Secur. 11:3077. doi: 10.3390/foods11193077, PMID: 36230153 PMC9563975

[ref23] Jiang A. Zhang Y. Zhang X. Wu D. Liu Z. Li S.. (2020). Morin alleviates LPS-induced mastitis by inhibiting the PI3K/AKT, MAPK, NF-κB and NLRP3 signaling pathway and protecting the integrity of blood-milk barrier. Int. Immunopharmacol. 78:105972. doi: 10.1016/j.intimp.2019.105972, PMID: 31711938

[ref24] Jingjing L. Lacey G. Richard H. Jun Y. (2009). Combined yeast-derived beta-glucan with anti-tumor monoclonal antibody for cancer immunotherapy. Exp. Mol. Pathol. 86, 208–214. doi: 10.1016/j.yexmp.2009.01.006 19454271 PMC2685877

[ref25] Jing-Jing W. Zheng-Kai W. Xu Z. Ya-Nan W. Yun-He F. Zheng-Tao Y. (2017). Butyrate protects against disruption of the blood-milk barrier and moderates inflammatory responses in a model of mastitis induced by lipopolysaccharide. Br. J. Pharmacol. 174, 3811–3822. doi: 10.1111/bph.13976 28800679 PMC5647178

[ref26] Jinjing W. Mengqi L. Feiyun Z. Chengtuo N. Chunfeng L. Qi L.. (2018). Cell wall polysaccharides: before and after autolysis of brewer’s yeast. World J. Microbiol. Biotechnol. 34:137. doi: 10.1007/s11274-018-2508-6, PMID: 30128783

[ref27] Karolina K.-S. Jakub R. Mateusz F. Marcin F. Wojciech M. (2020). *Saccharomyces boulardii* CNCM I-745: a non-bacterial microorganism used as probiotic agent in supporting treatment of selected diseases. Curr. Microbiol. 77, 1987–1996. doi: 10.1007/s00284-020-02053-9, PMID: 32472262 PMC7415030

[ref28] Kruttika D. Gustaf H. Suzanne D. (2019). The gut microbiome and metabolic syndrome. J. Clin. Invest. 129, 4050–4057. doi: 10.1172/JCI129194 31573550 PMC6763239

[ref29] Lei L. Caimei W. Daiwen C. Bing Y. Zhiqing H. Yuheng L.. (2020). Selenium-enriched yeast alleviates oxidative stress-induced intestinal mucosa disruption in weaned pigs. Oxidative Med. Cell. Longev. 2020:5490743. doi: 10.1155/2020/5490743, PMID: 32256952 PMC7106930

[ref30] Li J. Jin H. Yan X. Shao D. Hu X. Shi J. (2021). The anti-obesity effects exerted by different fractions of Artemisia sphaerocephala Krasch polysaccharide in diet-induced obese mice. Int. J. Biol. Macromol. 182, 825–837. doi: 10.1016/j.ijbiomac.2021.04.070, PMID: 33864863

[ref31] Lin C. Alexander C. Steelman A. Warzecha C. de Godoy M. Swanson K. (2019). Effects of a *Saccharomyces cerevisiae* fermentation product on fecal characteristics, nutrient digestibility, fecal fermentative end-products, fecal microbial populations, immune function, and diet palatability in adult dogs1. J. Anim. Sci. 97, 1586–1599. doi: 10.1093/jas/skz064, PMID: 30770927 PMC6447260

[ref32] Lv J. Guo L. Chen B. Hao K. Ma H. Liu Y.. (2022). Effects of different probiotic fermented feeds on production performance and intestinal health of laying hens. Poult. Sci. 101:101570. doi: 10.1016/j.psj.2021.101570, PMID: 34852968 PMC8639472

[ref9001] Meijuan M. Xu Z. Ran H. Xuerui L. Guangjun C. Xiangzhen S. (2023). Disodium fumarate alleviates endoplasmic reticulum stress, mitochondrial damage, and oxidative stress induced by the high-concentrate diet in the mammary gland tissue of Hu sheep. Antioxidants (Basel). 12. doi: 10.3390/antiox12020223 PMC9952365 36829784

[ref33] Meng Z. Dengpan B. Jiaqi W. Xiaoqiao Z. Dan Z. Ting Z.. (2015). Milk production and composition responds to dietary neutral detergent fiber and starch ratio in dairy cows. Anim. Sci. J. 87, 756–766. doi: 10.1111/asj.12482 26712573

[ref34] Miloš M. M. Milica G. P. Marija D. M. Branka I. O. Zorica S. S. (2019). Hematoprotective effects and antioxidant properties of β-glucan and vitamin C against acetaminophen-induced toxicity: an experimental study in rats. Drug Chem. Toxicol. 44, 302–309. doi: 10.1080/01480545.2019.1587451 30880499

[ref35] Ni J. Wu G. D. Albenberg L. Tomov V. (2017). Gut microbiota and IBD: causation or correlation? Nat. Rev. Gastroenterol. Hepatol. 14, 573–584. doi: 10.1038/nrgastro.2017.88, PMID: 28743984 PMC5880536

[ref36] Pedro F.-J. Gary W B. William C. Douwe V. S. Jose M.-M. (2023). Fungal β-glucan-facilitated cross-feeding activities between *Bacteroides* and *Bifidobacterium* species. Commun. Biol. 6:576. doi: 10.1038/s42003-023-04970-4, PMID: 37253778 PMC10229575

[ref9002] Pei Q. Lizhi W. (2024). Effect of adding yeast cultures to high-grain conditions on production performance, rumen fermentation profile, microbial abundance, and immunity in goats. Animals (Basel). 14. doi: 10.3390/ani14121799 PMC11200607 38929418

[ref37] Pittayanon R. Lau J. Yuan Y. Leontiadis G. Tse F. Surette M.. (2019). Gut microbiota in patients with irritable bowel syndrome-a systematic review. Gastroenterology 157, 97–108. doi: 10.1053/j.gastro.2019.03.049, PMID: 30940523

[ref38] Plaizier J. Krause D. Gozho G. McBride B. (2008). Subacute ruminal acidosis in dairy cows: the physiological causes, incidence and consequences. Vet. J. (London, England: 1997) 176, 21–31. doi: 10.1016/j.tvjl.2007.12.016, PMID: 18329918

[ref39] Qian W. Hao J. Hongli Z. Weiqiao L. Xiao W. Wenfeng X.. (2023). Β-Glucan-conjugated anti-PD-L1 antibody enhances antitumor efficacy in preclinical mouse models. Carbohydr. Polym. 324:121564. doi: 10.1016/j.carbpol.2023.121564 37985066

[ref40] Qinwen W. Qianyue Y. Xingyin L. (2023). The microbiota-gut-brain axis and neurodevelopmental disorders. Protein Cell 14, 762–775. doi: 10.1093/procel/pwad026 37166201 PMC10599644

[ref41] Qu C. Chen W. Zhihao Y. Liuping C. Yingdong N. Shengyong M.. (2021). Whole transcriptome analysis of RNA expression profiles reveals the potential regulating action of long noncoding RNA in lactating cows fed a high concentrate diet. Anim. Nutr. 7, 1315–1328. doi: 10.1016/j.aninu.2021.10.002, PMID: 34786504 PMC8567331

[ref42] Ruibo T. Wencheng Y. Jianhua S. Kaihe X. Shuang L. Caijun Z.. (2024). The rumen microbiota contributed to the development of mastitis induced by subclinical ketosis. Microb. Pathog. 187:106509. doi: 10.1016/j.micpath.2023.106509 38185451

[ref43] Runqi F. Chan L. Daiwen C. Gang T. Ping Z. Jun H.. (2023). Yeast hydrolysate attenuates lipopolysaccharide-induced inflammatory responses and intestinal barrier damage in weaned piglets. J. Anim. Sci. Biotechnol. 14:44. doi: 10.1186/s40104-023-00835-2, PMID: 36932457 PMC10021991

[ref44] Sales K. Reimer R. (2023). Unlocking a novel determinant of athletic performance: the role of the gut microbiota, short-chain fatty acids, and “biotics” in exercise. J. Sport Health Sci. 12, 36–44. doi: 10.1016/j.jshs.2022.09.002, PMID: 36089243 PMC9923434

[ref45] Sanders M. E. Merenstein D. J. Reid G. Gibson G. R. Rastall R. A. (2019). Probiotics and prebiotics in intestinal health and disease: from biology to the clinic. Nat. Rev. Gastroenterol. Hepatol. 16:642. doi: 10.1038/s41575-019-0199-6, PMID: 31399728

[ref46] Seon-Kyun K. Robin G. B. You-Tae K. Joongi K. Hyeri K. Jae Hyoung C.. (2019). Role of probiotics in human gut microbiome-associated diseases. J. Microbiol. Biotechnol. 29, 1335–1340. doi: 10.4014/jmb.1906.06064 31434172

[ref9003] Shendong Z. Jie H. Hao Z. Xiaokun S. Yijin J. Xu Z. (2025). Live yeast (Saccharomyces cerevisiae) improves growth performance and liver metabolic status of lactating Hu sheep. J. Dairy. Sci. 108. doi: 10.3168/jds.2024-25829 39986452

[ref47] Siyu W. Cheng W. Qifan Z. Hui Y. Edward C D. Xin Z.. (2022). Dynamics of microbial communities during inulin fermentation associated with the temporal response in SCFA production. Carbohydr. Polym. 298:120057. doi: 10.1016/j.carbpol.2022.120057 36241315

[ref48] Song Y. Loor J. J. Li C. Liang Y. Li N. Shu X.. (2021). Enhanced mitochondrial dysfunction and oxidative stress in the mammary gland of cows with clinical ketosis. J. Dairy Sci. 104, 6909–6918. doi: 10.3168/jds.2020-19964, PMID: 33715853

[ref49] Spencer C. N. McQuade J. L. Gopalakrishnan V. McCulloch J. A. Vetizou M. Cogdill A. P.. (2021). Dietary fiber and probiotics influence the gut microbiome and melanoma immunotherapy response. Science 374, 1632–1640. doi: 10.1126/science.aaz7015, PMID: 34941392 PMC8970537

[ref50] Stefanska B. Człapa W. Pruszynska-Oszmałek E. Szczepankiewicz D. Fievez V. Komisarek J.. (2017). Subacute ruminal acidosis affects fermentation and endotoxin concentration in the rumen and relative expression of the CD14/TLR4/MD2 genes involved in lipopolysaccharide systemic immune response in dairy cows. J. Dairy Sci. 101, 1297–1310. doi: 10.3168/jds.2017-12896 29153518

[ref51] Tao Z. Yingyu M. Ruiyang Z. Yanfeng X. Changzheng G. Wangpan Q.. (2022). Responsive changes of rumen microbiome and metabolome in dairy cows with different susceptibility to subacute ruminal acidosis. Anim. Nutr. 8, 331–340. doi: 10.1016/j.aninu.2021.10.009, PMID: 35024470 PMC8718735

[ref52] Tian T. Mao Q. Xie J. Wang Y. Shao W. Zhong Q.. (2022). Multi-omics data reveals the disturbance of glycerophospholipid metabolism caused by disordered gut microbiota in depressed mice. J. Adv. Res. 39, 135–145. doi: 10.1016/j.jare.2021.10.002, PMID: 35777903 PMC9263645

[ref53] Ting Y. Chenyu W. Lifen L. Xuan X. Hui Z. Wentao Z.. (2024). Effects of fermented sweet potato residue on nutrient digestibility, meat quality, and intestinal microbes in broilers. Anim. Nutr. 17, 75–86. doi: 10.1016/j.aninu.2024.03.007 38737580 PMC11087712

[ref54] Vivek C. G. Mais K. Christopher B. J. Eamonn M. M. Q. M P. Alexander F. C. Efficacy of probiotics in irritable bowel syndrome: systematic review and meta-analysis. Gastroenterology 165, 1206–1218. doi: 10.1053/j.gastro.2023.07.018 37541528

[ref55] Wang Y. Du W. Hu X. Yu X. Guo C. Jin X.. (2023). Targeting the blood-brain barrier to delay aging-accompanied neurological diseases by modulating gut microbiota, circadian rhythms, and their interplays. Acta Pharm. Sin. B 13, 4667–4687. doi: 10.1016/j.apsb.2023.08.009, PMID: 38045038 PMC10692395

[ref56] Wenjin G. Juxiong L. Wen L. He M. Qian G. Xingchi K.. (2020). Niacin alleviates dairy cow mastitis by regulating the GPR109A/AMPK/NRF2 signaling pathway. Int. J. Mol. Sci. 21:3321. doi: 10.3390/ijms21093321, PMID: 32397071 PMC7246865

[ref57] Wen-Yang C. Li-Jen L. Yun-Chen H. Shen-Chang C. Tzu-Tai L. (2020). Effects of *Saccharomyces cerevisiae* and phytase co-fermentation of wheat bran on growth, antioxidation, immunity and intestinal morphology in broilers. Anim. Biosci. 34, 1157–1168. doi: 10.5713/ajas.20.0399 33152224 PMC8255880

[ref58] Wu K. Shang S. Bao L. Zhao Y. Guan Z. Xu J.. (2023). Retinoic acid ameliorates low-grade endotoxemia-induced mastitis by limiting inflammatory responses in mice. Microb. Pathog. 185:106426. doi: 10.1016/j.micpath.2023.106426, PMID: 37879450

[ref59] Xiaoyu H. Jian G. Caijun Z. Peng J. T M. Li Y.. (2020). The gut microbiota contributes to the development of *Staphylococcus aureus*-induced mastitis in mice. ISME J. 14, 1897–1910. doi: 10.1038/s41396-020-0651-1 32341472 PMC7305118

[ref60] Xiaoyu H. Shuang L. Ruiying M. Jian G. Caijun Z. Yongguo C.. (2022). The rumen microbiota contributes to the development of mastitis in dairy cows. Microbiol. Spectr. 10:e0251221. doi: 10.1128/spectrum.02512-21 35196821 PMC8865570

[ref61] Xiaoyu H. Zhaoqi H. Caijun Z. Yuhong H. Min Q. Kaihe X.. (2023). Gut/rumen-mammary gland axis in mastitis: gut/rumen microbiota-mediated “gastroenterogenic mastitis”. J. Adv. Res. 55, 159–171. doi: 10.1016/j.jare.2023.02.009 36822391 PMC10770137

[ref62] Yang W. C. Chen H. Y. Tzu-Tai L. (2020). The effects of fungal feed additives in animals: a review. Animals (Basel) 10:894. doi: 10.3390/ani10050894, PMID: 32384791 PMC7278461

[ref63] Yuan L. Chang J. Yin Q. Lu M. Di Y. Wang P.. (2017). Fermented soybean meal improves the growth performance, nutrient digestibility, and microbial flora in piglets. Anim. Nutr. (Zhongguo xu mu shou yi xue hui) 3, 19–24. doi: 10.1016/j.aninu.2016.11.003, PMID: 29767125 PMC5941061

[ref64] Yuanhang S. Jianying L. Minqiang S. Yaokun L. Yongqing G. Guangbin L.. (2023). A study on differential biomarkers in the milk of Holstein cows with different somatic cells count levels. Animals (Basel) 13:2446. doi: 10.3390/ani13152446 37570255 PMC10417570

[ref65] Yue W. Yiguang Z. Xiangfang T. Xuemei N. Linshu J. Hui W.. (2024). Nutrition, gastrointestinal microorganisms and metabolites in mastitis occurrence and control. Anim. Nutr. 17, 220–231. doi: 10.1016/j.aninu.2024.01.010 38800734 PMC11126769

[ref66] Zhang Y. Wang X. Lin J. Liu J. Wang K. Nie Q.. (2024). A microbial metabolite inhibits the HIF-2α-ceramide pathway to mediate the beneficial effects of time-restricted feeding on MASH. Cell Metab. 36, 1823–1838.e6. doi: 10.1016/j.cmet.2024.07.004, PMID: 39079531

[ref67] Zhe W. Zhong W. Tangsheng L. Wenhao C. Wei Y. Kai Y.. (2022). The microbiota-gut-brain axis in sleep disorders. Sleep Med. Rev. 65:101691. doi: 10.1016/j.smrv.2022.101691 36099873

[ref68] Zhen-Ping Z. Yue D. Ting-Ting F. Ying Z. Bang-Ce Y. (2024). Biomarker-responsive engineered probiotic diagnoses, records, and ameliorates inflammatory bowel disease in mice. Cell Host Microbe 31, 199–212.e5. doi: 10.1016/j.chom.2022.12.004, PMID: 36758520

[ref69] Zhenyu W. Yu B. Yu P. Walter J. J. G. Sonja D. V. Lijun S.. (2021). Xylan alleviates dietary fiber deprivation-induced dysbiosis by selectively promoting *Bifidobacterium pseudocatenulatum* in pigs. Microbiome 9:227. doi: 10.1186/s40168-021-01175-x, PMID: 34802456 PMC8606072

[ref70] Zhi-Yuan L. Lin F. Wei-Dan J. Pei W. Yang L. Jun J.. (2022). Dietary mannan oligosaccharides strengthens intestinal immune barrier function via multipath cooperation during *Aeromonas hydrophila* infection in grass carp (*Ctenopharyngodon idella*). Front. Immunol. 13:1010221. doi: 10.3389/fimmu.2022.1010221 36177013 PMC9513311

